# Distinct roles of the *YPEL* gene family in development and pathogenicity in the ascomycete fungus *Magnaporthe oryzae*

**DOI:** 10.1038/s41598-018-32633-6

**Published:** 2018-09-27

**Authors:** Joon-Hee Han, Jong-Hwan Shin, Yong-Hwan Lee, Kyoung Su Kim

**Affiliations:** 1 Division of Bio-Resource Sciences and Bioherb Research Institute, Kangwon National University, Chuncheon, 24341 Korea; 2 Department of Agricultural Biotechnology and Center for Fungal Genetic Resources, Seoul National University, Seoul, 08826 Korea

## Abstract

Members of the *Yippee*-*like* (*YPEL*) gene family are highly conserved in eukaryotes and are homologous to the *Drosophila yippee* gene. In this study, we functionally characterized two *YPEL*-homologous genes, *MoYPEL1* and *MoYPEL2*, in the rice blast pathogen *Magnaporthe oryzae* using the deletion mutants *ΔMoypel1*, *ΔMoypel2*, and *ΔΔMoypel1*,*2*. The *MoYPEL1* deletion mutant was significantly defective in conidiation and unable to undergo appressorium development; however, deletion of *MoYPEL2* resulted in a significant increase in conidiation and the abnormal development of two appressoria per conidium. These data demonstrate the opposite roles of each member of the *YPEL* gene family during the development of *M*. *oryzae*. The double mutant was phenotypically similar to the *ΔMoypel1* mutant in conidiation, but similar to the *ΔMoypel2* mutant in appressorium development. Subcellular localization of the MoYPEL1 protein was dynamic during appressorium development, while the MoYPEL2 protein consistently localized within the nuclei during developmental stages. Our studies indicate that the two *YPEL* gene family members play distinct roles in the developmental stages of *M*. *oryzae*, furthering our understanding of disease dissemination and development in fungi.

## Introduction

Rice blast caused by the ascomycete fungal pathogen *Magnaporthe oryzae* is a threat to global rice production. This disease continues to be important with increased global population size and food demand^1,2^. In addition, the pathogen has a remarkable ability to destroy the whole rice plant including the leaf, collar, node, panicle base (neck), panicle, and sometimes root, unlike some pathogens that infect plants in a tissue-specific manner^3^. *Magnaporthe oryzae* is a polycyclic pathogen that enables the production of massive amounts of conidia (asexual spores) through multiple rounds of reproduction during the rice growing season. The nature of the pathogen implies a higher potential for epidemic incidence. The *M*. *oryzae* conidium develops a specialized infection structure called the appressorium at the tip of the conidial germ tube upon recognition of plant surface signals^4^. Generation of turgor pressure in the appressorium allows direct penetration of the plant epidermal cells. Following colonization of host cells, *M*. *oryzae* reproduces asexually to produce conidia that serve as a major dispersal unit and inoculum. Therefore, understanding the molecular events of conidiation and subsequent conidium-mediated disease development is important for developing novel strategies for plant protection.

*Magnaporthe oryzae* has served as a model for the study of the molecular events underlying fungal development, pathogenicity, and interaction with host plants. These cellular events are achieved by complex processes in which signaling pathways play a critical role in the genetic regulation and cross-talk with other signaling cascades for fungal development and pathogeneicty^5^. The cyclic AMP (cAMP)-associated protein kinase A signaling is known to regulate surface recognition and pathogenicity of *M*. *oryzae*^6^. A well-conserved mitogen-activated protein kinase (MAPK) pathway regulates appressorium development and subsequent invasive growth inside host plant cells in *M*. *oryzae*^7–9^. Glycogen and lipid mobilization to the appressorium is dependent on PMK1, a MAPK orthologous yeast Fus3/Kss1, following which glycogen and lipids are degraded via the protein kinase A pathway for turgor generation^9^. Mps1, a MAPK orthologous to yeast Slt2, is essential for cell wall integrity, appressorium penetration and invasive growth in *M*. *oryzae*^10,11^. Precise regulation of the cell cycle is critical, especially during infection-related development of *M*. *oryzae*, as in multicellular organisms^12,13^. The entry into mitosis and cell cycle arrest following mitotic entry and invasion of plant cells, respectively, are essential for appressorium differentiation^14,15^. Further investigation uncovered that two independent S-phase cell cycle checkpoints are required for appressorium-mediated plant infection in *M*. *oryzae*^16^. Recently, it was shown that CDC14 in *M*. *oryzae* (*MoCDC14*), which is orthologous to the phosphatase CDC14 in *Saccharomyces cerevisiae*^17,18^, plays a key role in septation and nuclear distribution, which are linked to the proper regulation of appressorium formation, conidiation, and growth in *M*. *oryzae*^19^.

In this study, we set out to elucidate the roles of the members of the *Yippee-like* (*YPEL*) gene family in the development and pathogenicity of *M*. *oryzae*, which are homologous to the first identified *Drosophila* Yippee gene coding for a protein with a putative zinc finger-like metal binding domain^20^. The YPEL family, which consists of one member in yeast and five members in mammals, is highly conserved across eukaryotic taxa^21,22^. Members of the *YPEL* gene family have been shown to be associated with various cellular processes including the cell cycle, senescence, and development in mammals^23–25^. Recently, the *MOH1* gene, which is orthologous to the *Drosophila* Yippee protein, was shown to be involved in apoptosis induced by DNA damage in *S*. *cerevisiae*^26^. However, the YPEL family remains uncharacterized in filamentous fungi, which prompted us to investigate the functional roles of the phytopathogenic fungus *M*. *oryzae*. Our analysis *in silico* led to the identification of two *YPEL* genes (named *MoYPEL1* and *MoYPEL2*) in *M*. *oryzae*, unlike in yeasts, which have only one. Here, we investigated the functional roles of the *MoYPEL1* and *MoYPEL2* genes during *M*. *oryzae* morphogenesis and disease development through targeted gene deletion. Our study indicates important and distinct roles of the *YPEL* gene family in disease dissemination and development in the phytopathogenic fungus *M*. *oryzae*.

## Results

### Phylogenetic analysis of MoYPEL proteins

**Figure 1:**
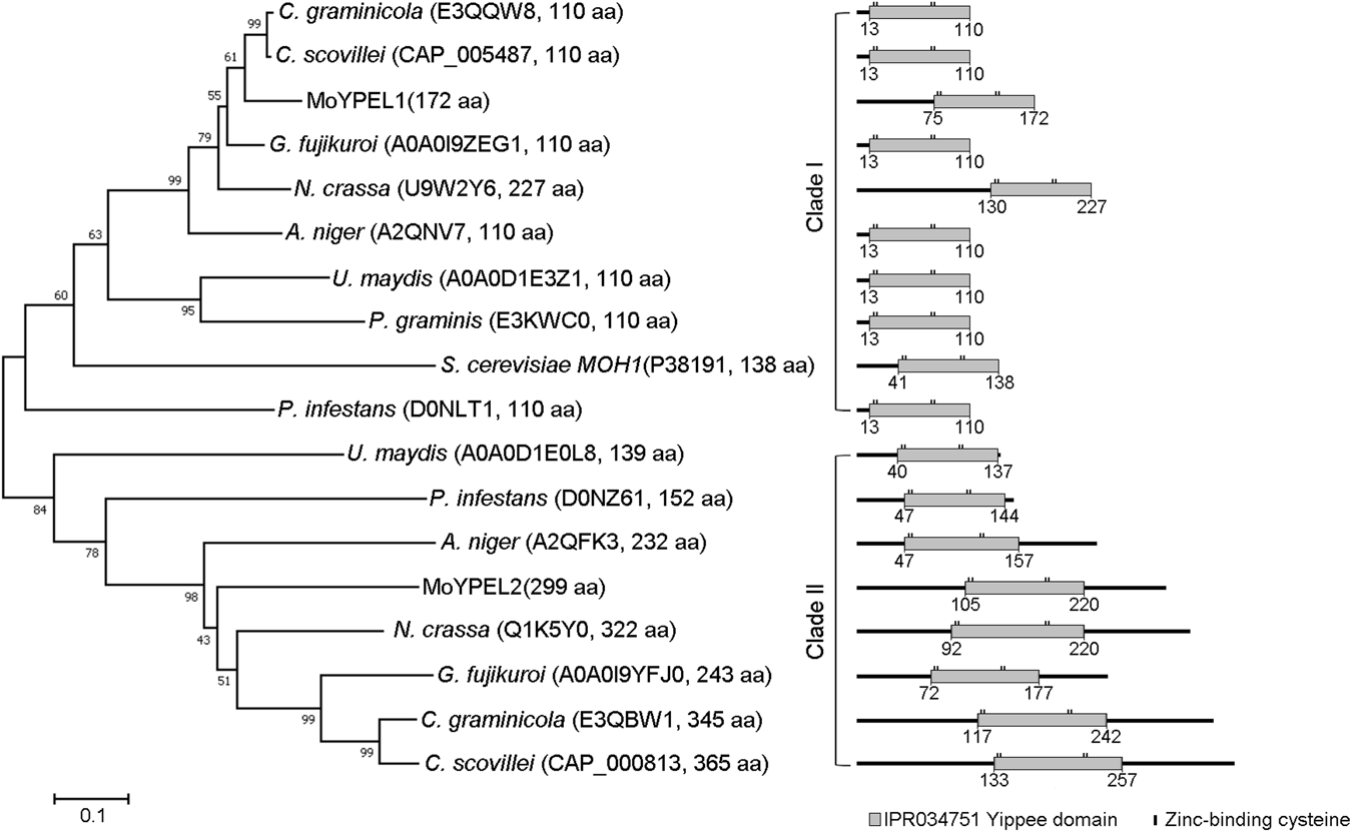
Phylogenetic analysis of MoYPEL proteins. A neighbor-joining tree was derived from the alignment of amino acid sequences of related proteins in fungal taxa. Numbers at nodes represent the percentage of the occurrence in 1,000 bootstrap replicates. Scale bar indicates the number of amino acid differences per site. Numbers indicate positions of amino acids for the Yippee domain. GenBank accession number and amino acid length are in parentheses followed by species. Gray box represents the YPEL domain with information regarding amino acid positions and the zinc-binding cysteine pocket.

**Figure 2:**
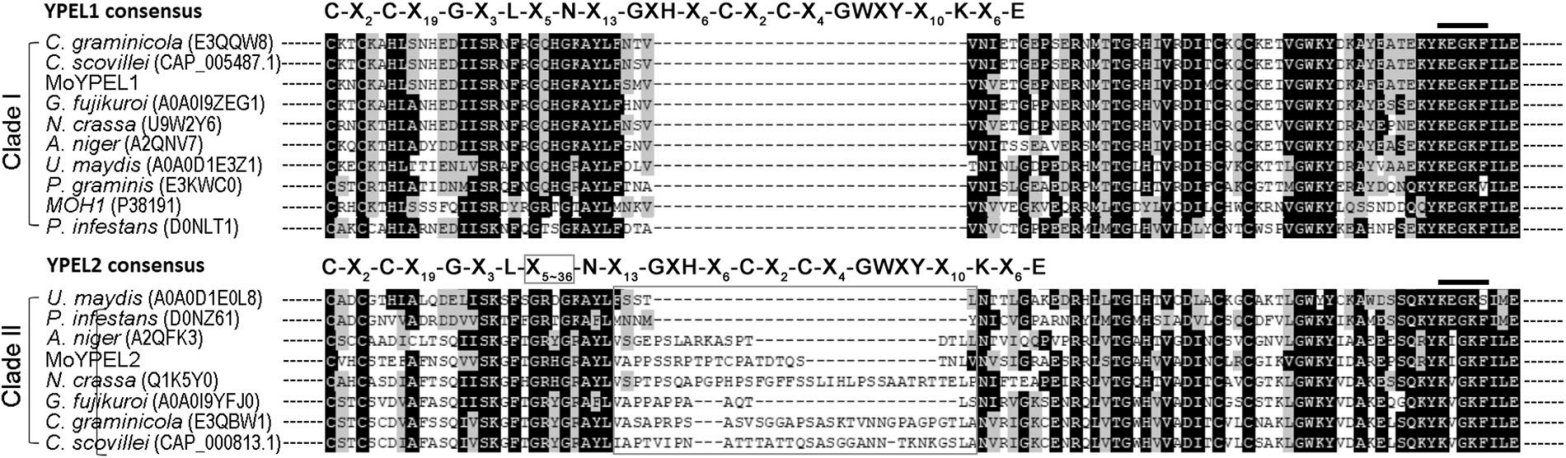
Amino acid sequence alignment of YPEL proteins. Line indicates the nuclear localization signal at the C-terminus of the proteins. Note that the YPEL1 consensus sequence in clade I is consistent with the previously reported consensus sequence VII and the YPEL2 consensus sequence in clade II has a sequence expansion (boxed) at the X_5_ position in the consensus sequence VII. Accession numbers of proteins are shown in Fig. 1.

*Magnaporthe oryzae* was found to contain two *YPEL* genes based on BLAST search of the genome database (http://fungi.ensembl.org) using conserved sequences (IPR034751) of the Yippee protein. The resulting sequences were then aligned with the sequences of the five human YPEL genes and the yeast MOH1 gene to reveal phylogenetic relationships (Supplementary Fig. S1)^22,26^. Based on phylogenetic analysis, the two *M*. *oryzae* genes were named *MoYPEL1* (MGG_06263.7) and *MoYPEL2* (MGG_00255.7). MoYPEL1 forms a clade together with human YPEL5 and yeast MOH1 with 42% and 49% sequence identities, respectively (Supplementary Table S1). MoYPEL2 forms a separate lineage with the remaining human YPELs, while it shares 42% and 40% identity with the human YPEL4 and MoYPEL1, respectively. Phylogenetic analysis of MoYPELs and related proteins in fungal taxa showed that MoYPEL1 forms a separate lineage within ascomycetes in which it shares the highest identity of 88% with the E3QQW8 protein of *Colletotrichum graminicola* (Fig. 1 and Supplementary Table S1). In clade I, MoYPEL1 and the other related proteins are distantly related to yeast MOH1. MoYPEL2 shares relatively low similarity with ascomycete proteins, among which, 55% identity with the E3QBW1 protein of *C*. *graminicola* was the highest value. We found that filamentous ascomycetes have two YPEL-related proteins, unlike *S*. *cerevisiae*. The two separate lineages and low similarity between MoYPEL1 and MoYPEL2 may imply potentially different functions of the two proteins in the cellular events of *M*. *oryzae*. Alignment of YPEL-related proteins revealed that homologs of MoYPEL1 have consensus sequences, which is consistent with consensus sequence VII (C-X_2_-C-X_19_-G-X_3_-L-X_5_-N-X_13_-GXH-X_6_-C-X_2_-C-X_4_-GWXY-X_10_-K-X_6_-E) reported in a previous study (Fig. 2)^20^. MoYPEL2-related proteins in clade II also have an additional sequence expansion that is variable from X_5_ to X_36_ at position X_5_ of consensus sequence VII. All of the fungal YPEL proteins contain a nuclear localization signal (NLS) at the C-terminus of this domain.

### Subcellular localization of MoYPEL proteins

**Figure 3:**
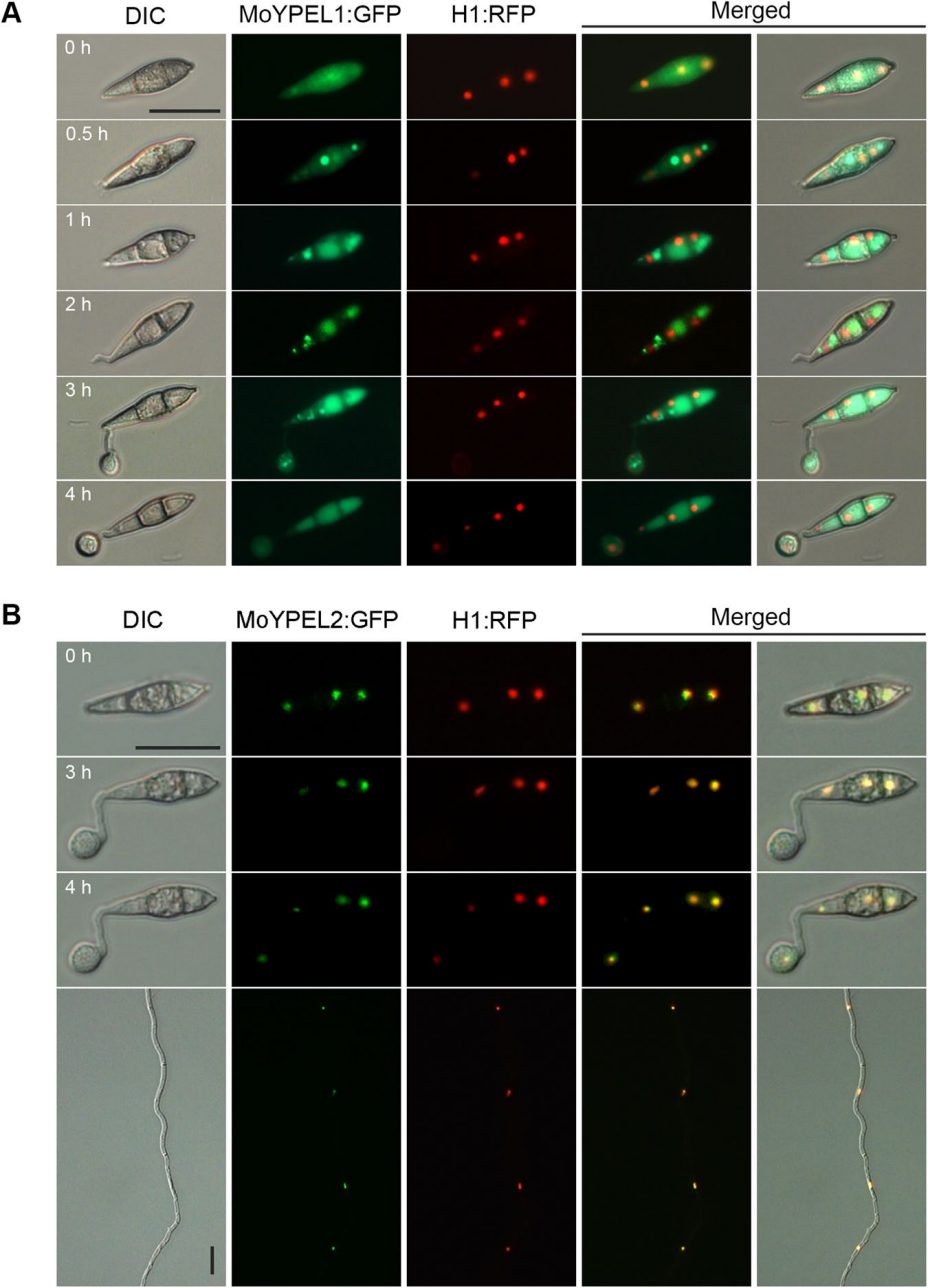
Subcellular localization of MoYPEL protein in *M*. *oryzae*. (**A**) A dynamic change in MoYPEL1:sGFP localization. Briefly, the MoYPEL1:sGFP fusion protein was initially localized in conidium cytoplasm, followed by aggregation next to nuclei and diffuse distribution in the cytoplasm. Newly aggregated MoYPEL1:sGFP protein emerged at both sides of the nucleus during germ tube development. An aggregated form of MoYPEL1:sGFP protein subsequently appeared in the appressorium initial, and disappeared following nuclear division during appressorium development. Conidia were observed on coverslips at the indicated time points during appressorium development. (**B**) Localization of MoYPEL2:sGFP. The obvious colocalization (orange) of MoYPEL2:sGFP and histone HI:RFP fusion proteins was observed during appressorium development and hyphal growth. Scale bars = 20 µm.

To further understand the potential cellular roles of MoYPEL proteins, we generated transformants expressing MoYPEL1:sGFP and MoYPEL2:sGFP fusion proteins by transforming protoplasts of a transformant expressing a histone H1:RFP fusion protein^27,28^. The transformants were confirmed to be phenotypically similar to wild type (Supplementary Table S2). The MoYPEL1:sGFP fusion protein was initially distributed uniformly in the cytoplasm and nuclei of conidia (Fig. 3A). Interestingly, a marked increase in aggregation of the MoYPEL1 signal was quickly observed next to nuclei in conidium cells, which were subsequently distributed diffusely in the cytoplasm next to nuclei. As a germ tube emerged, the MoYPEL1 signal translocated out of the nucleus as assembled particles at the third conidium cell. Prior to mitosis, the assembled forms of the MoYPEL1 signal were maintained not only in the third cell, but also in an appressorium initial. Finally, the aggregated MoYPEL1 signal in the appressorium was diffusely distributed similar to the distribution in conidium cells following mitosis. Unlike the dynamic localization of the MoYPEL1:sGFP signal during appressorium development, the MoYPEL2:sGFP signal colocalized stably with nuclei not only during vegetative growth, but also during appressorium development (Fig. 3B). The differing subcellular localizations of the MoYPEL proteins suggest their distinctly different roles during *M*. *oryzae* morphogenesis and disease development.

### Roles of the *MoYPEL* genes in vegetative growth

**Figure 4:**
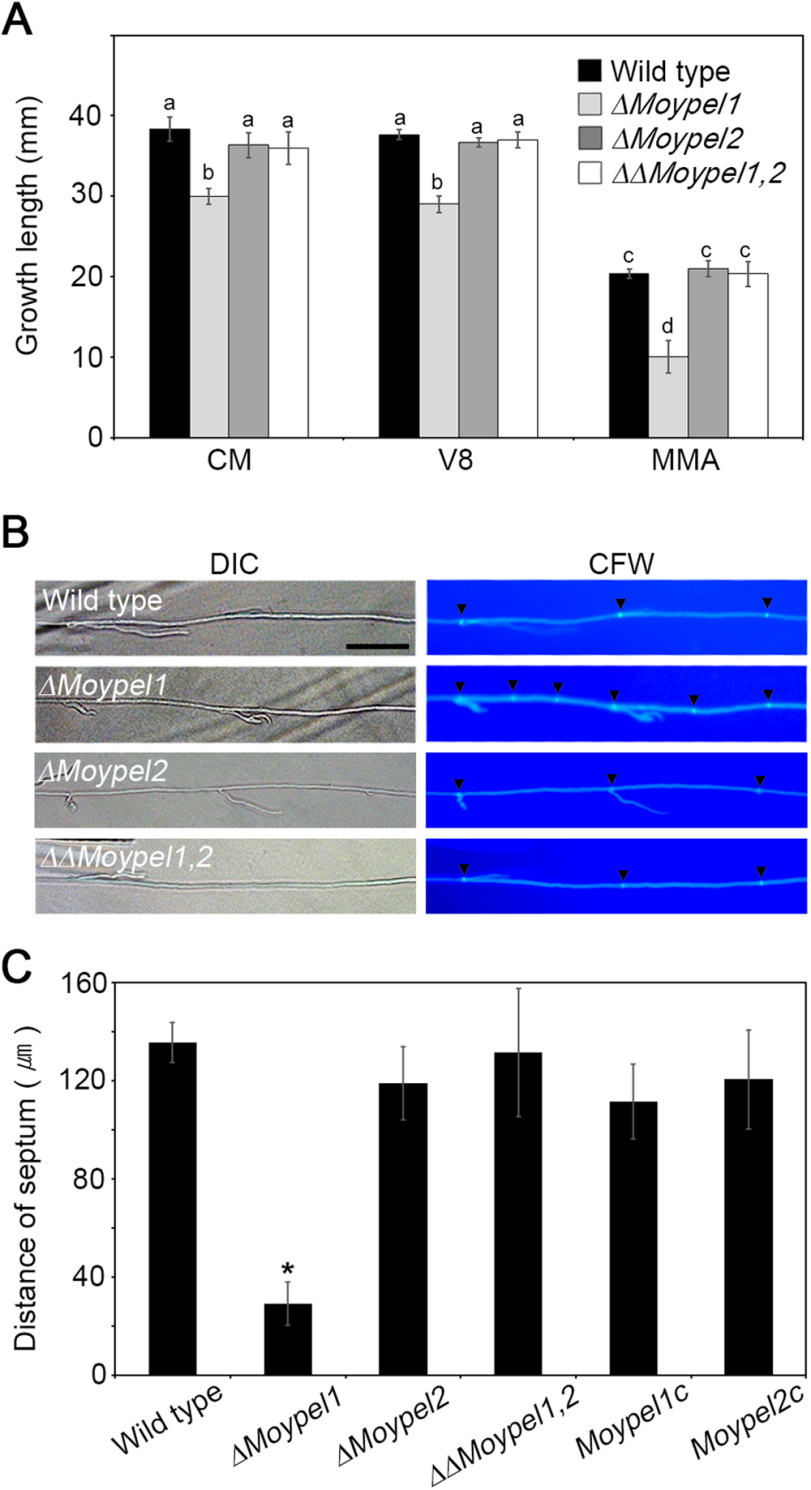
Role of *MoYPEL* genes in *M*. *oryzae* vegetative growth. (**A**) A defect of the *ΔMoypel1* mutant in vegetative growth. Measurement of vegetative growth was conducted on plates containing different media using inoculated mycelial plugs obtained from water agar culture plates. Data were presented as means ± SD from three independent experiments with three replicates per experiment. Different letters on bars indicate significant differences according to Tukey’s test at *p* < 0.05. (**B**) Increased septation during vegetative growth of the *ΔMoypel1* mutant. Septation was visualized in hyphae at 16 h post-inoculation on complete medium with calcofluor white staining. Scale bar = 30 µm. (**C**) Hyphal compartments were reduced in size in the *ΔMoypel1* mutant. Hyphal compartment size was measured using ZEN imaging software. At least 50 hyphal compartments per strain were measured (n = 52, 56, 54, and 52 for wild-type, *ΔMoypel1*, *ΔMoypel2*, and *ΔΔMoypel1*,*2* respectively).

To determine the functional role of *MoYPEL* genes in growth, we measured vegetative growth on solid media including complete medium (CM), V8 medium, and minimal medium (MM). The *ΔMoypel1* mutant exhibited a significant defect on all three nutrient conditions while the growth of *ΔMoypel2* was indistinguishable from that of the wild type (Fig. 4A). The vegetative growth of the double mutant *ΔΔMoypel1*,*2* was also similar to that of the wild type. Compared to the growth of the wild type, growth of the *ΔMoypel1* mutant was reduced by about 23% on CM and V8 medium, and more severely by 50% on MM. To further investigate the reduced vegetative growth in the *ΔMoypel1* mutant, hyphae of the mutant were stained with calcofluor white (CFW) to test septation and hyphal morphology during vegetative growth. Unlike the wild type and other mutants, increased septation was observed in the *ΔMoypel1* mutant (Fig. 4B), although the hyphal morphology of the mutants was similar to that of the wild type. The average distance of the hyphal compartments of the *ΔMoypel1* mutant was 29.3 µm, while the wild type, *ΔMoypel2*, and *ΔΔMoypel1*,*2* strains were 135, 118.9, and 131.4 µm, respectively, as shown in Fig. 4C. This result suggests that the reduced vegetative growth was due to shortened hyphal compartments, which was a result of the increased septation in the *ΔMoypel1* mutant.

### Roles of the *MoYPEL* genes in conidiation

**Figure 5:**
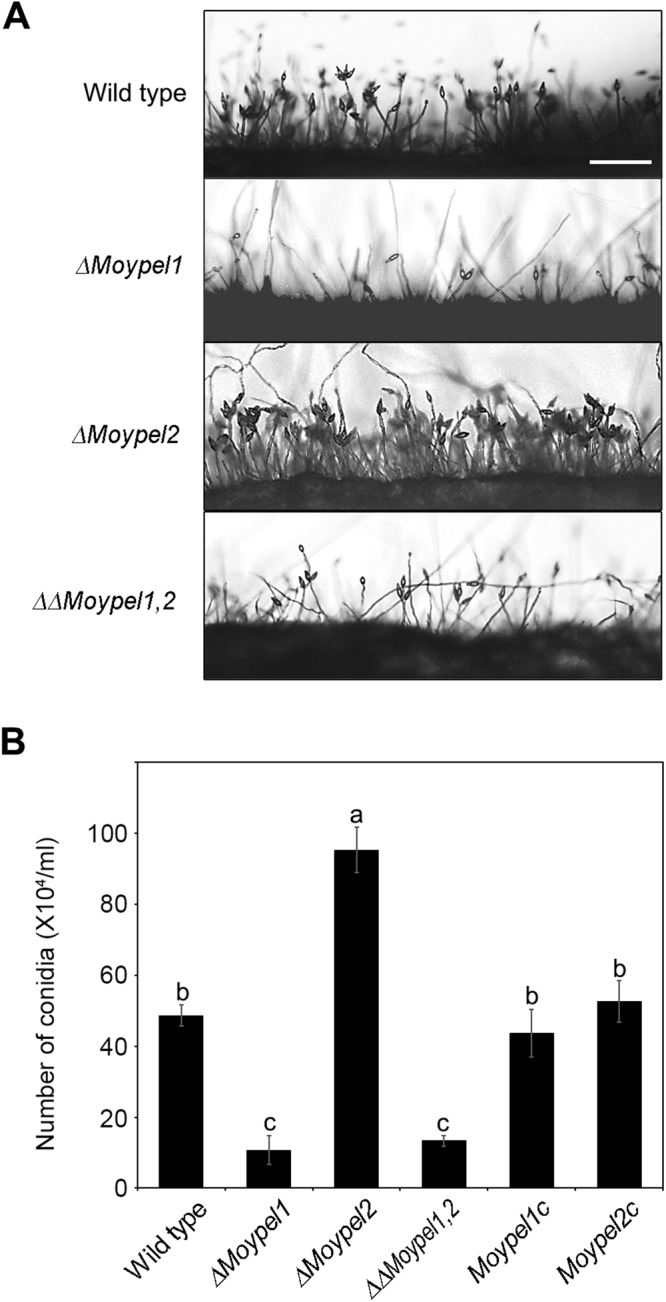
Different roles of the *MoYPEL* genes in *M*. *oryzae* conidiation. (**A**) Microscopic visualization of conidiation. Note the dramatic reduction in conidiation in the *ΔMoypel1* and *ΔΔMoypel1*,*2* mutants, but an increase in the *ΔMoypel2* mutant. Scale bar = 100 µm. (**B**) Quantitative measurement of conidia. Conidia were collected from 7-day-old V8 culture plates with 5 ml of distilled water. Data were presented as means ± SD from three independent experiments with three replicates per experiment (n ≥ 100 conidia per strain). Different letters on bars indicate significant differences according to Tukey’s test at *p* < 0.05.

Because conidia serve as a major inoculum and propagule in many fungal pathogens, we assessed association of the *MoYPEL* genes with conidiation by measuring the production of conidia. Microscopic observation revealed that the *ΔMoypel1* and *ΔΔMoypel1*,*2* mutants showed considerably impaired ability to produce conidia compared with that of the wild type (Fig. 5A). However, the *ΔMoypel2* mutant showed an increased ability with more dense conidia. Quantitative measurement of conidium production indicated that the *ΔMoypel1* and *ΔΔMoypel1*,*2* mutants produced significantly less conidia by 22% and 27%, respectively, but the *ΔMoypel2* mutant produced significantly more conidia by 151%, compared with the wild type (Fig. 5B). Such abnormality of the *ΔMoypel1* and *ΔMoypel2* mutants was rescued in the complemented transformants *Moypel1c* and *Moypel2c*, respectively. These results suggest that each of *MoYPEL* genes plays a distinct and opposite role in the regulation of conidiation.

**Figure 6:**
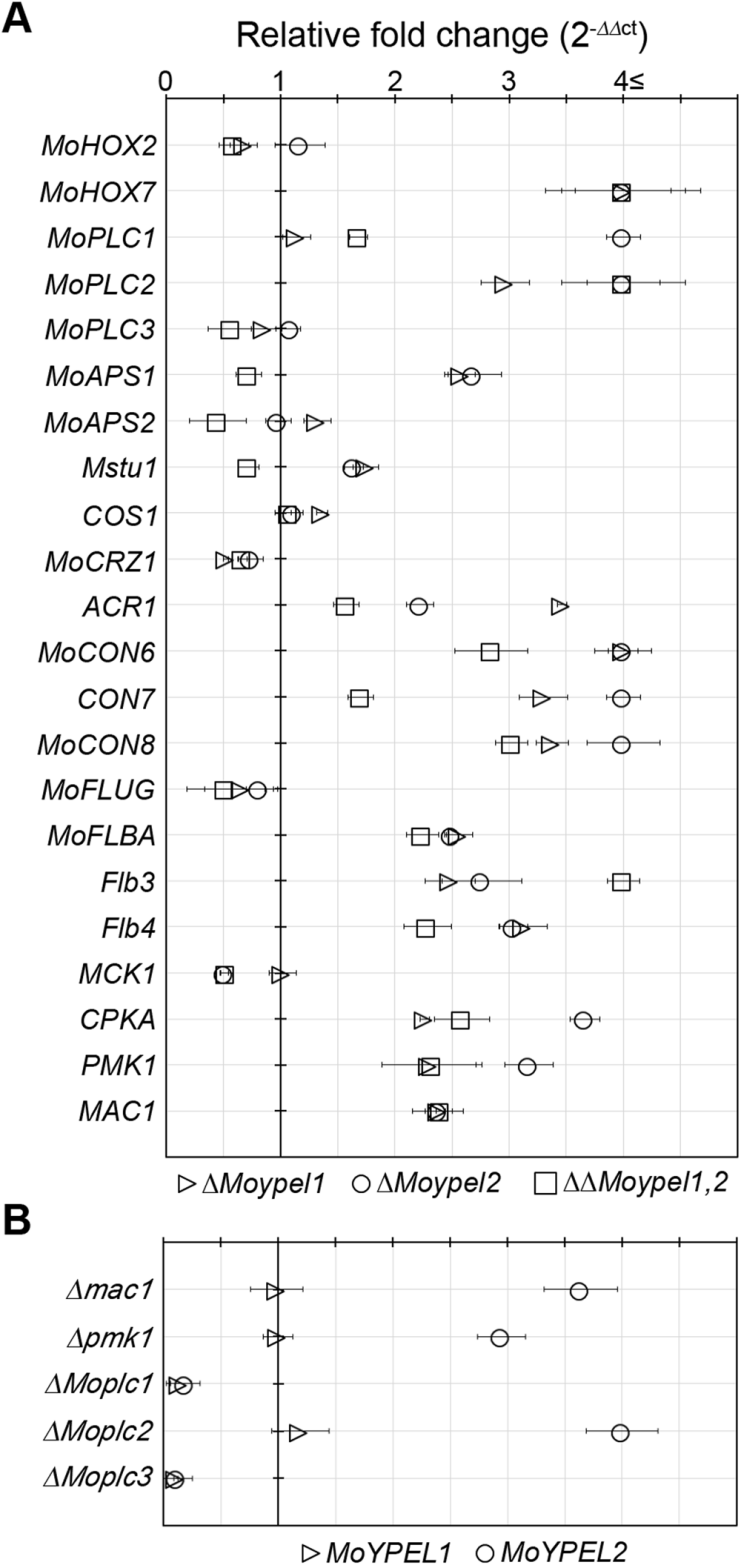
Measurement of gene expression changes by quantitative RT-PCR analysis. (**A**) Expression levels of genes in *MoYPEL* deletion mutants. The transcript abundance of the indicated genes in each deletion mutant was normalized to *β-tubulin* and expressed relative to a value of 1 in the wild type KJ201. (**B**) Expression of *MoYPEL* genes in various deletion mutants. Mutant information is included in Table 2. The transcript abundance of each *MoYPEL* gene in each mutant strain is expressed relative to a value of 1 in the wild type KJ201. Total RNAs were extracted from purified conidia grown in oatmeal agar plates for 10 days.

To examine the effect of *MoYPEL* deletion on the expression of genes important for conidiation in *M*. *oryzae*, we measured gene expression in each deletion mutant using quantitative RT-PCR (qRT-PCR) (Table 1). Differences in gene expression were observed in each deletion mutant (Fig. 6A). Unlike the expression of *MoPLC3*, *MoPLC1* was significantly upregulated only in the *ΔMoypel2* mutant, and *MoPLC2* expression was upregulated in the *ΔMoypel1*, *ΔMoypel2*, and *ΔΔMoypel1*,*2* mutants. Transcripts of *MoAPS1*, *ACR1*, *MoCON6*, *CON7*, *MoCON8*, *MoFLBA*, *Flb3*, and *Flb4* were significantly increased at different levels in both the *ΔMoypel1* and *ΔMoypel2* mutants, among which the *MoCON6*, *MoCON8*, *MoFLBA*, *Flb3*, *and Flb4* genes were also upregulated in the *ΔΔMoypel1*,*2* mutant. The *CPKA*, *PMK1*, and *MAC1* genes were highly expressed in the *ΔMoypel1*, *ΔMoypel2*, *and ΔΔMoypel1*,*2* mutants. The different levels of gene expression in the *ΔMoypel1* and *ΔMoypel2* mutants suggest that the *MoYPEL1* and *MoYPEL2* genes play distinct roles in *M*. *oryzae* conidiation.

**Table 1:** *Magnaporthe oryzae* genes used in quantitative real-time PCR.

Gene	Locus No.	Descriptions	Reference
*MoHOX2*	MGG_00184	Homeobox TF, no condiation	Kim *et al*.^48^
*MoHOX7*	MGG_12865	Homeobox TF, no appressorium formation	Kim *et al*.^48^
*MoPLC1*	MGG_02444	Phospholipase C gene, infection-related development and pathogenicity	Rho *et al*.^32^
*MoPLC2*	MGG_05332	Phospholipase C gene, reduced conidiation and defect in appressorium	Choi *et al*.^31^
*MoPLC3*	MGG_08315	Phospholipase C gene, reduced conidiation and defect in appressorium	Choi *et al*.^31^
*MoAPS1*	MGG_09869	APSES TF, reduced conidiation	Park *et al*.^49^
*MoAPS2*	MGG_08463	APSES TF, reduced conidiation	Park *et al*.^49^
*Mstu1*	MGG_00692	APSES TF, reduced conidiation and mycelial growth, defect in appressorium	Nishimura *et al*.^50^
*COS1*	MGG_03394	C_2_H_2_ zinc finger TF, conidiophores stalk-less	Zhou *et al*.^51^
*MoCRZ1*	MGG_05133	A calcineurin-responsive TF, reduced conidiation and pathogenicity, abnormal appreossorium	Choi *et al*.^37^
*ACR1*	MGG_09847	Hypothetical protein, acropetal conidia	Lau & Hamer^52^
*MoCON6*	MGG_02246	Hypothetical protein, ortholog to *con-6* in *Neurospora crassa*	Madi *et al*.^53^
*CON7*	MGG_05287	C_2_H_2_ zinc finger TF, abnormal conidia	Odenbach *et al*.^54^
*MoCON8*	MGG_00513	Hypothetical protein, ortholog to *con-8* in *N*. *crassa*	Madi *et al*.^53^
*MoFLUG*	MGG_02538	Putative glutamine synthetase, ortholog to *fluG* in *Aspergiluus nidulans*	Lee & Adams^55^
*MoFLBA*	MGG_14517	Putative regulator of G protein signaling, ortholog to *flbA* in *A*. *nidulans*	Wieser *et al*.^56^
*Flb3*	MGG_04699	C_2_H_2_ zinc finger TF, aerial mycelium formation in *M*. *oryzae*, ortholog to *flbC* in *A*. *nidulans*	Wieser *et al*.^56^, Matheis *et al*.^57^
*Flb4*	MGG_06898	Myb TF, no conidiation, ortholog to *flbD* in *A*. *nidulans*	Wieser *et al*.^56^, Matheis *et al*.^57^
*MCK1*	MGG_00883	Protein kinase, reduced conidiation and appressorium development unable to penetrate plant tissues	Jeon *et al*.^11^
*CPKA*	MGG_06368	cAMP-dependent protein kinase, defect in penetration	Xu *et al*.^58^
*PMK1*	MGG_09565	A MAP kinase gene, defects in appressorium formation and invasive growth	Xu & Hamer^59^
*MAC1*	MGG_09898	Adenylate cyclase, reduced vegetative growth, conidiation, and conidial germination, defects in appressorium formation and penetration	Choi & Dean^60^

To understand the impact of signaling pathways on the expression of *MoYPEL* genes, we measured expression of the *MoYPEL* genes in signaling pathway-related mutant backgrounds (Table 2 and Fig. 6B). The *Δmac1* mutant lacks an adenylate cyclase, the *Δpmk1* mutant lacks an MAPK, and the *ΔMoplc2* mutant lacks phospholipase C. *MoYPEL2* was significantly induced in all three mutants, although the expression of *MoYPEL1* was almost unchanged in these mutants. Both *MoYPEL* genes were significantly downregulated in the phospholipase C mutants *ΔMoplc1* and *ΔMoplc3*, but not in the *ΔMoplc2* mutant, which supports the different roles of the *MoPLC* genes, as previously reported^23^. This suggests that the regulation of *MoYPEL* expression may be associated with Ca^2+^ and cAMP-dependent signaling pathways.

**Table 2:** Various fungal strains used in this study.

Strains	Genotypes	Reference
KJ201	Wild type	This study
*ΔMoYPEL1*	*MoYPEL1* deletion mutant of KJ201	“
*MoYPEL1c*	Complemented transformant of *ΔMoYPEL1* mutant	“
*MoYPEL1:GFP*	MoYPEL1:GFP and H1:RFP tagged strain	“
*ΔMoYPEL2*	*MoYPEL2* deletion mutant of KJ201	“
*MoYPEL2c*	Complemented transformant of *ΔMoYPEL2* mutant	“
*MoYPEL2:GFP*	MoYPEL2:GFP and H1:RFP tagged strain	“
*ΔΔMoypel1*,*2*	*MoYPEL1* and *MoYPEL2* double deletion mutant	“
*Δmac1*	*MAC1* deletion mutant of 70–15	Choi & Dean^60^
*Δpmk1*	*PMK1* deletion mutant of Guy11	Xu & Hamer^59^
*ΔMoplc1*	*MoPLC1* deletion mutant of 70–15	Rho *et al*.^32^
*ΔMoplc2*	*MoPLC2* deletion mutant of KJ201	Choi *et al*.^31^
*ΔMoplc3*	*MoPLC3* deletion mutant of KJ201	Choi *et al*.^31^

### Roles of the *MoYPEL* genes in appressorium development

**Figure 7:**
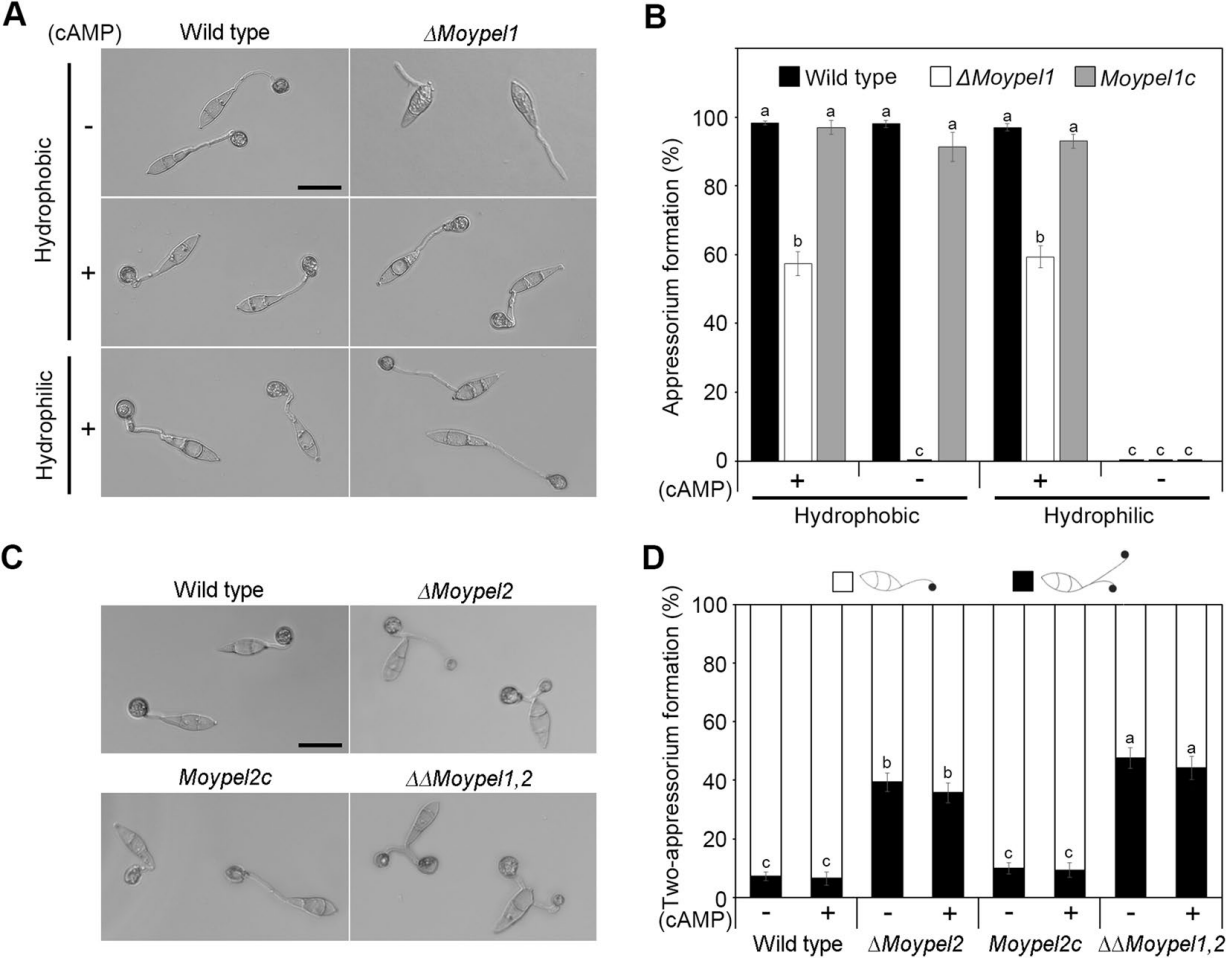
Role of *MoYPEL* genes in *M*. *oryzae* appressorium development. (**A**) The defect in appressorium development of the *ΔMoypel1* mutant. Appressorium development was induced on coverslips with or without exogenous cAMP treatment (5 µM) and observed after 8 h. Scale bar = 30 µm. (**B**) Quantitative measurement of appressoria. The number of appressoria was counted after 8 h with ZEN imaging software. (**C**) Abnormal appressorium development of the *ΔMoypel2* and *ΔΔMoypel1*,*2* mutants. Conidia were placed on the hydrophobic surface of coverslips, and observed after 8 h. Scale bar = 30 µm. (**D**) Quantitative comparison of abnormal appressoria on a hydrophobic surface with or without exogenous cAMP treatment (5 µM). The number of appressoria was counted after 72 h with ZEN imaging software. Data were presented as means ± SD from three independent experiments with three replicates per experiment. Different letters on bars indicate significant differences according to Tukey’s test at *p* < 0.05.

As appressorium-mediated penetration plays a key role in *M*. *oryzae*, we tested the ability of the tested strains to develop appressoria upon sensing an inductive hydrophobic surface. This experiment revealed that *ΔMoypel1* was defective in appressorium development on the hydrophobic surface, while almost all conidia from wild type and *Moypel1c* developed appressoria (Fig. 7A,B). Next, we investigated the effect of exogenous cAMP on appressorium development of the *ΔMoypel1* mutant.

Almost all conidia from wild type developed appressoria on both hydrophobic and hydrophilic surfaces with treatment of 5 μM exogenous cAMP. Treatment with exogenous cAMP markedly restored appressorium formation in the *ΔMoypel1* mutant; Approximately 57.3% and 59.3% of the *ΔMoypel1* conidia formed appressoria on the inductive and non-inductive surfaces, respectively, with exogenous cAMP treatment (Fig. 7A,B). These results indicate that the *ΔMoypel1* mutant may be defective in sensing signals of hydrophobic surface and intracellular cAMP signaling pathway for appressorium development. Unlike the defect of *ΔMoypel1* in appressorium development, the *ΔMoypel2* mutant produced a large number of instances of two appressoria on two separated germ tubes emerged from conidia (Fig. 7C). The different sizes of the two appressoria in the *ΔMoypel2* mutant are indicative of the subsequent development of two appressoria. Similar to the *ΔMoypel2* mutant, the *ΔΔMoypel1*,*2* mutant also produced two appressoria. At 48 h, 10.3% and 16.3% of the *ΔMoypel2* and *ΔΔMoypel1*,*2* mutants, respectively, formed two appressoria compared with 2.3% and 2.0% of the wild type and *Moypel2c* (data not shown). During prolonged incubation (72 h), these percentages increased dramatically to 39.3% and 47.7% of the *ΔMoypel2* and *ΔΔMoypel1*,*2* mutants, respectively (Fig. 7D). The percentage of two appressoria in *ΔMoypel2* and *ΔΔMoypel1*,*2* was unaffected with treatment of exogenous cAMP. This result suggests that *MoYPEL2* plays a distinct role in the regulation of appressorium development, given that a defect of *ΔMoypel1* mutant in appressorium development is recovered with exogenous cAMP treatment.

Consistent with the roles of *MoYPEL* genes in appressorium development, changes in transcription of appressorium-related genes were detected as shown in Fig. 6A. These include the *MoHOX7*, *MoPLC2*, *CPKA*, *PMK1*, and *MAC1* genes, which were significantly upregulated at different levels in the *ΔMoypel1*, *ΔMoypel2*, and *ΔΔMoypel1*,*2* mutants, although *MoPLC1* was highly upregulated only in the *ΔMoypel2* mutant. In addition, expression changes of the *MoYPEL* genes in the signaling pathway-related mutant backgrounds (Table 2 and Fig. 6B) suggest the possibility that the Ca^2+^ and cAMP-dependent signaling pathways are linked to *MoYPEL*-mediated appressorium development.

### Roles of the *MoYPEL* genes in pathogenicity

**Figure 8:**
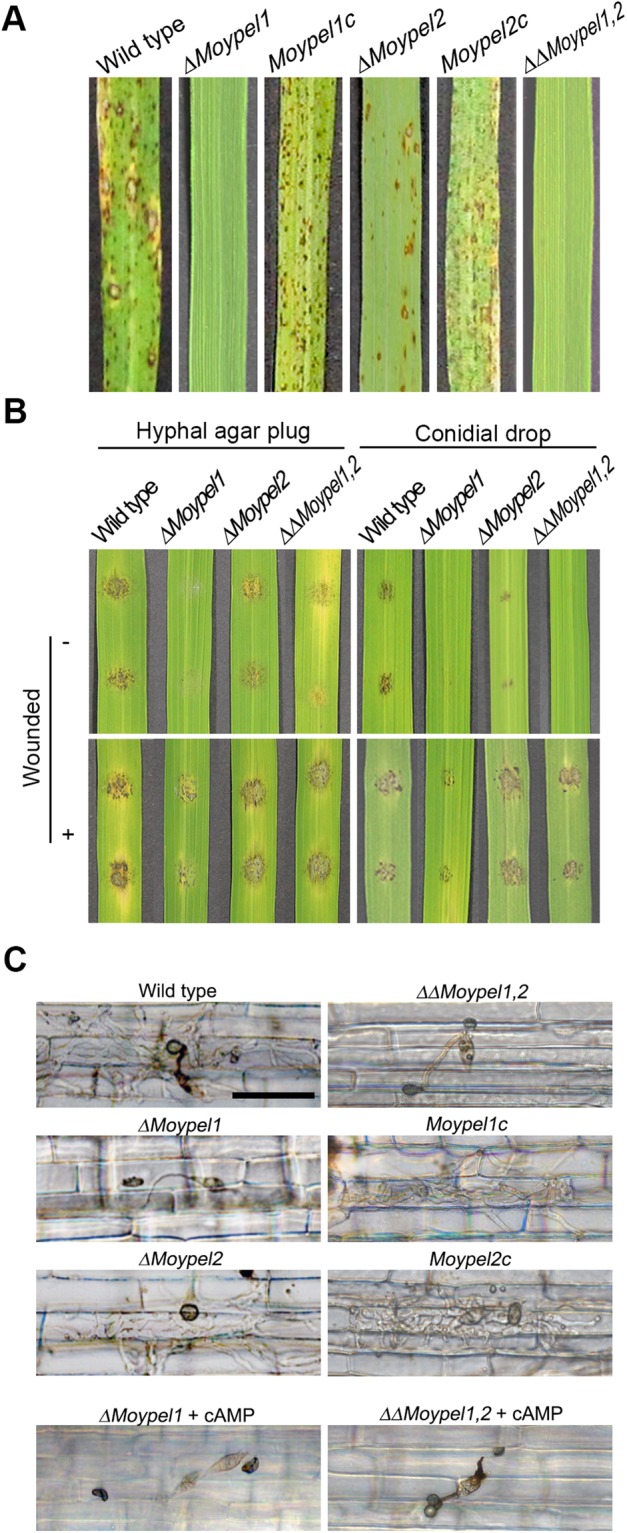
Role of *MoYPEL* genes in *M*. *oryzae* pathogenicity. (**A**) Spray assay on rice seedlings. Conidial suspension (5 × 10^5^ conidia ml^−1^) of indicated strains was sprayed on 3-week-old rice plants. Photographs were taken 7 days post-inoculation. (**B**) Infection assay on rice leaf. Hyphal agar plugs (5 mm) and drops (20 µl) of conidial suspension were placed on leaves with or without wounds and incubated for 4 days. (**C**) Penetration and invasion assay on rice sheath cells. Drops of conidial suspension (2 × 10^4^ conidia ml^−1^) were inoculated with or without the addition of exogenous cAMP (5 µM) on rice sheath cells and incubated for 48 h. Scale bar = 50 µm.

To evaluate the involvement of the *MoYPEL* genes in pathogenicity, conidial suspension was sprayed onto susceptible rice seedlings (cv. Nakdongbyoe). The wild type caused severe necrotic lesions, but the *ΔMoypel1* and *ΔΔMoypel1*,*2* mutants were non-pathogenic (Fig. 8A). The *ΔMoypel2* mutant appeared slightly less severe than the wild type. The defects of the mutants were rescued in the corresponding complemented transformants. To further characterize the role of *MoYPEL* genes in pathogenicity, we inoculated hyphal agar plugs and conidial drops on both intact and wounded sites of detached leaflets of the rice cultivar. Consistent with the spray assay, inoculation of hyphal plugs and conidial drops from the wild type and the *ΔMoypel2* mutant strains developed severe lesions, but no disease symptoms were observed with the *ΔMoypel1* mutant on intact sites. The *ΔΔMoypel1*,*2* mutant resulted in fewer reduced lesions from hyphal agar plugs on intact sites, but not from the conidial drops (Fig. 8B). Unlike the inoculations on intact sites, the wounded inoculations of both hyphal plugs and conidial drops from all strains developed severe disease symptoms. This result suggests that the failure of the *ΔMoypel1* and *ΔΔMoypel1*,*2* mutants with conidium inoculation are due to an inability to penetrate, rather than a defect in the ability to undergo invasive growth inside host cells. When rice sheath cells were inoculated with conidial suspension, the wild type and *ΔMoypel2* mutant developed appressoria and exhibited subsequent invasive growth (Fig. 8C). In contrast, the *ΔMoypel1* mutant was able to develop appressoria but unable to undergo appressorium-mediated penetration and invasive growth on rice sheath cells. A further investigation of pathogenic development of *ΔMoypel1* and *ΔΔMoypel1*,*2* mutants on rice sheath cells with treatment of exogenous cAMP had no effect on the defects of the two mutants in appressorium-mediated penetration (Fig. 8C). These results indicate that *MoYPEL1* is essential for appressorium-mediated penetration, possibly, independent of cAMP-related pathway, and *MoYPEL2* is necessary for the full virulence in *M*. *oryzae*. The complemented transformants of these mutants were successful in penetration and invasive growth.

Because the *ΔMoypel1* mutant developed appressoria on rice sheath cells, but not on artificial hydrophobic surface (Fig. 7), we tested host factors inducing appressorium development by exogenously adding two known host inducers, a cutin monomer, 1,16-hexadecanediol and a primary alcohol, 1-octacosanol on both hydrophilic and hydrophobic surfaces^29,30^. Compared to those of the wild type and *Moypel1*c, the *ΔMoypel1* mutant was still defective in appressorium development in the treatment of 10 µM 1,16-hexadecanediol (Fig. S3). However, 54.3% and 37% of conidia in *ΔMoypel1* mutant developed appressoria on the hydrophobic and hydrophilic surfaces, respectively, with the treatment of 10 mM 1-octacosanol, suggesting that the *ΔMoypel1* mutant is partially able to response to certain host signals such as a primary alcohol, 1-octacosanol present on host cells. These results indicate that *MoYPEL1* is involved in sensing certain physical and chemical signals such as hydrophobicity and a cutin monomer for appressorium development.

## Discussion

Although the *YPEL* gene family exists in all eukaryotes, functional roles of *YPEL* genes have been mostly uncharacterized in eukaryotic organisms, including filamentous phytopathogenic fungi^22^. This prompted us to elucidate the detailed roles of *MoYPEL* genes in the pathogenic development of the rice blast pathogen *M*. *oryzae*. In this study, filamentous fungi, unlike yeast, were revealed to contain two *YPEL* genes^26^. Our phylogenetic analyses revealed the distant relatedness of fungal *YPEL* genes with the yeast *MOH1* gene, which is homologous to the human *YPEL5* gene, raising the possibility that the fungal *YPEL* genes play different roles than the apoptotic role of *MOH1*^26^. Given that each member of a gene family has evolved toward divergence in sequence and function, separate lineage formation of the fungal *YPEL* genes would support functional differences of the different members of the fungal *YPEL* gene family. Our findings support the conclusion that each member of the *YPEL* gene family plays distinctly different roles in *M*. *oryzae* development and pathogenicity.

The disparate subcellular localization patterns of MoYPEL:sGFP fusion proteins are intriguing. Stable nuclear localization of the MoYPEL2 fusion protein during different developmental stages of *M*. *oryzae* was consistent with the presence of an NLS at the C-terminus. In addition, this expression pattern may suggest the constitutive expression of the MoYPEL2 protein at the translational level. Unlike MoYPEL2 localization, subcellular localization of the MoYPEL1:sGFP fusion protein was dynamic during appressorium development. MoYPEL1 proteins were initially distributed in both the cytoplasm and nuclei of conidia. Subsequent subcellular translocation of MoYPEL1 protein appears associated with appressorium development. During germ tube and appressorium formation, the MoYPEL1 protein translocated out of the cytoplasm and nucleus as assembled particles, which were maintained until mitosis. Given that YPEL5, which is homologous to MoYPEL1, is involved in cell cycle progression in monkey cells^21^, it has been speculated that MoYPEL1 may be involved in the cell cycle during appressorium development, until further investigation shows MoYPEL1 to colocalize with cellular organelles in mitosis.

To characterize the functional roles of *MoYPEL* genes, we generated the knockout mutants *ΔMoypel1*, *ΔMoypel2*, and *ΔΔMoypel1*,*2*, which exhibit pleiotropic defects in morphogenesis and disease development in *M*. *oryzae*. Notably, the *MoYPEL* gene family was shown to be important especially for disease dissemination and development in *M*. *oryzae*. Conidium production was highly reduced in the *ΔMoypel1* mutant, but increased in the *ΔMoypel2* mutant, indicating the opposite regulation of *MoYPEL* genes in conidium production in *M*. *oryzae*. Interestingly, the double mutant *ΔΔMoypel1*,*2* was similar to the *ΔMoypel1* mutant in conidium production, suggesting that *MoYPEL1* is downstream of *MoYPEL2* in the proper regulation of conidium production. Detailed biochemical functions of YPEL proteins remain unknown in eukaryotes including fungi. However, a zinc-binding motif (two cysteine pairs) and NLS motifs of MoYPEL proteins imply potential roles in protein-protein interactions and transcriptional regulation. The nuclear localization of MoYPEL proteins and transcriptional changes of several conidiation and/or appressorium-related genes in *MoYPEL* mutants may reflect the involvement of MoYPEL proteins in transcription regulation. Interestingly, the *MoYPEL* genes were significantly changed in deletion mutants of signaling regulators. Unlike *MoYPEL1*, *MoYPEL2* was significantly upregulated in the three mutants *Δmac1*, *Δpmk1*, *ΔMoplc2*, suggesting that the regulation of *MoYPEL2* expression is associated with cAMP, MAPK, and Ca^2+^-dependent signaling pathways. However, a significant downregulation of the two *MoYPELs* in *ΔMoplc1* and *ΔMoplc2* mutants may suggest the interrelated but different roles of *MoPLC* genes not only in *MoYPEL* expression but also development regulation in *M*. *oryzae*, as previously suggested^31,32^. The subcellular translocation of MoYPEL1 from nuclei and the cytoplasm during appressorium development may also reflect the interaction of MoYPEL1 with unknown protein(s) involved in MoYPEL1 translocation and function during appressorium development. In animal COS-7 cells, YPEL5 has been shown through coimmunoprecipitation to physically interact with Ran-binding protein in the microtubule-organizing center (RanBPM)^21^.

Appressorium development is a key event for disease development in *M*. *oryzae*. *MoYPEL* genes was proven to be important for this process. The *ΔMoypel1* mutant was defective in sensing the hydrophobic surface for appressorium development, which was restored by exogenous cAMP treatment, indicating that such defect could be bypassed with complemented appressorium development of the *ΔMoypel1* mutant on both inductive and non-inductive surfaces. The fact that the *ΔMoypel1* mutant formed appressoria on rice sheath cells, as not observed on the hydrophobic surface, is an indicative of its ability to recognize a host inducer of appressorium development. Our subsequent assays revealed that the *ΔMoypel1* mutant partially recognized a primary alcohol, 1-octacosanol, but not a cutin monomer, 1,16-hexadecanediol. Taken together, these results indicate that *MoYPEL1* was involved in sensing chemical and physical signal cues for appressorium development in *M*. *oryzae*. Unlike the *ΔMoypel1* mutant, abnormal development of two appressoria per conidium in the *ΔMoypel2* mutant indicates the opposite role in appressorium development in *M*. *oryzae*. The phenotype of the *ΔMoypel2* mutant is similar to those of deletion mutants for *MoPLC2* and *MoPLC3*, as shown in our previous study^31^. Considering that the expression of *MoYPEL* genes is modulated in deletion mutants of *MoPLC* genes, functional roles of *MoYPEL* genes could be associated with Ca^2+^-mediated regulation of appressorium development. As expected, the *ΔMoypel1* and *ΔΔMoypel1*,*2* mutants were non-pathogenic due to their inability to penetrate plant cell walls and initiate invasive growth. However, the *ΔMoypel2* mutant developed a slight reduction in disease symptoms following penetration and invasive growth in plant cells. Collectively, our study demonstrates that *MoYPEL* genes play important roles in growth, conidiation, and appressorium development in *M*. *oryzae*.

## Methods

### Fungal strains and culture conditions

*Magnaporthe oryzae* wild type strain KJ201 and fungal transformants generated in this study were routinely cultured on oatmeal agar plates (OMA; 5% oatmeal and 1.5% agar powder) or V8 agar plates (V8; 8% V8 juice and 1.5% agar powder) for 10 days at 25 °C, under constant fluorescent light to promote conidiation. Mycelia used for genomic DNA and total RNA extraction were prepared by growing the relevant strains in liquid CM (0.6% yeast extract, 0.6% casamino acids, and 1% sucrose) for 3 days at 25 °C with agitation (150 rpm), or directly obtained from TB3 agar medium (0.3% yeast extract, 0.3% casamino acids, 1% glucose, 20% sucrose, and 0.8% agar powder).

### Preparation of protoplasts

*Magnaporthe oryzae* mycelia were cultured in liquid CM for 2–3 days and harvested with a bottle top filter, as described by Parsons *et al*.^33^. Briefly, mycelia were washed twice and resuspended in 20% sucrose. Lysing enzymes from *Trichoderma harzianum* (Sigma-Aldrich, St. Louis, MO, USA) was added to the mycelial suspension (5 mg/ml). The protoplasts were separated from the mycelia by filtration through two layers of Miracloth (Calbiochem, La Jolla, CA, USA). After centrifugation of the protoplast suspension at 5,000 rpm for 15 min at 4 °C, the protoplast pellet was twice washed with STC buffer (20% sucrose, 0.05 M Tris/Cl pH 8.0, and 0.05 M CaCl_2_), and re-suspended after centrifugation with STC buffer to make a final concentration of 5 × 10^7^ protoplasts ml^−1^.

### Bioinformatics tools

The protein sequences were obtained from the National Center for Biotechnology Information (NCBI); homology search of protein sequences was performed using the BLAST algorithm. Domain architectures were drawn using InterProScan software^34^. The phylogenetic tree was generated with a neighbor-joining method with 1,000 bootstrap replicates with MEGA7 software (http://www.megasoftware.net)^35^. Amino acid sequences were aligned using BioEdit Ver. 7.0.5 software (http://www.mbio.ncsu.edu/BioEdit/bioedit.html).

### Vector construction and fungal transformation

To investigate the functional roles of *MoYPEL* genes, we generated the targeted gene knockout mutants *ΔMoypel1* and *ΔMoypel2* using homology-dependent gene replacement as illustrated in Figure S2. Targeted deletion constructs for *MoYPEL1* and *MoYPEL2* was made by a modified double joint PCR^36^. Each fragment corresponding to 1.5 kb upstream and downstream of *MoYPEL1* and *MoYPEL2* was amplified with primers 5F/5R and 3F/3R (Supplementary Table S3), respectively. A 1.5-kb HPH cassette was amplified with the primers HPHF/HPHR from pBCATPH, which contains the hygromycin phosphotransferase gene^37^. The three PCR products were fused by rounds of fusion PCR, and the final deletion construct was amplified with the nested primers NF/NR. Protoplast suspension (5 × 10^7^ protoplasts ml^−1^) was used for a polyethylene glycol-mediated transformation of *M*. *oryzae*^38,39^. Briefly, the transformation was conducted by incubating 0.2 ml of protoplasts with DNA (2–3 µg the deletion construct) for 30 min at room temperature followed by the addition of 1 ml of PTC (40% PEG, 20% sucrose, 0.05 M Tris/Cl pH 8.0, 0.05 M CaCl_2_). The contents were mixed with 3 ml of TB3 (20% sucrose, 1% glucose, 0.3% yeast extract) regeneration medium. The mixture was plated TB3 agar plates supplemented with hygromycin (200 ppm). Hygromycin-resistant transformants were screened by PCR with the primers SF/SR. For the selected transformants, genomic DNA was extracted using the quick and safe method^40^. Targeted deletion was confirmed with Southern blot hybridization and RT-PCR. For complementation, a genomic copy of the targeted gene was amplified from wild type genomic DNA using the primers cmF/cmR. The amplicon was used for co-transforming protoplasts of the deletion mutant with the pII99 vector containing a geneticin-resistance cassette^41^. Complementation mutants were selected on media supplemented with geneticin (400 ppm) and screened for restoration of wild type phenotypes. The double knockout was amplified up- and downstream of *MoYPEL1* with the primers 5F/5R-Gen and 3F-Gen/3R, respectively. A 1.8-kb G418 cassette was amplified with the primers Gen_F/Gen_R from pII99, which contains the geneticin gene. The double knockout mutant *ΔΔMoypel1*,*2* was generated by co-transforming protoplasts of *ΔMoypel2* with the deletion construct for *MoYPEL1* and a geneticin cassette for selection.

### Nucleic acid manipulation

Fungal genomic DNA was used for PCR and Southern blot hybridization. Genomic DNA was isolated using two different methods. Genomic DNA for general experiments including restriction enzyme digestion, agarose gel separation, and DNA gel blotting was isolated according to a standard method^42^. Genomic DNA was digested with a restriction enzyme, *Sac*I, *Nco*I or *Sma*I, and blots were probed with a 500-base pair 5′ flanking cassette. DNA hybridization probes were labeled using Biotin-High Prime (Roche, Indianapolis, IN, USA) according to the manufacturer’s instructions. Chemiluminescent signal was detected using ChemiDoc XRS + system with Quantity One software (Bio-Rad Laboratories, Hercules, CA, USA).

### Localization of MoYPEL:sGFP fusion proteins

The MoYPEL:sGFP fusion vectors were generated by overlap cloning. PCR products of 2.6 and 2.9 kb, which included the 2.0 kb of the 5′-flanking promoter region and the open reading frame region of the *MoYPEL1* and *MoYPEL2* genes, were amplified with the primers YPEL1_F/YPEL1_R and YPEL2_F/YPEL2_R (Supplementary Table S3), respectively, from wild type KJ201 genomic DNA. A total of 5.2 kb of the sGFP gene including the HPH gene was amplified with the primers pIG-YPEL1_F/pIG-YPEL1_R and pIG-YPEL2_F/pIG-YPEL2_R from pIGPAPA^43^. The YPEL PCR products containing the promoter region and the 5.2-kb region of pIGPAPA including the sGFP and HPH cassettes were cloned using the overlap DNA Cloning Kit (Elpis Biotech, Taejeon, Korea). Each MoYPEL:sGFP fusion vector was introduced into the transformant expressing the histone H1:RFP fusion protein by transformation^27,28^. Fluorescence microscopy images were captured with a Carl Zeiss Axio Image A2 microscope (Carl Zeiss Microscope Division, Oberkochen, Germany).

### RNA isolation and expression analysis

For RT-PCR and qRT-PCR, total RNA was isolated from mycelia and conidia, respectively, using the Easy-Spin RNA extraction kit (iNtRON Biotechnology, Seongnam, Korea). First-strand complementary DNA (cDNA) was synthesized from total RNA using the SuperScript III First-strand Synthesis System (Invitrogen, Carlsbad, CA, USA) with oligo (dT) primers. RT-PCR was performed in a 20-μl reaction containing 10 ng of cDNA, 2.5 mM dNTPs, 2 μl of 10× PCR buffer, 1 μl (10 pmol) of each primer, and 1 unit of *Taq* polymerase. In all, 30 RT-PCR cycles were run on the Thermal Cycler Thermal Controller 2720 (Applied Biosystems, Foster City, CA, USA). The *β*-tubulin gene was included as a control. qRT-PCR reactions were performed as previously described^44^. The qRT-PCR mixture (final volume 10 μl) comprised 5 μl of Real-Time PCR 2× Master Mix (Elpis, Daejeon, Korea), 3 μl of forward and reverse primers (10 pmol of each), and 2 μl of cDNA template (25 ng μl^−1^). PCR was performed using a StepOne Real-Time PCR System (Applied Biosystems) as follows: 3 min at 95 °C (one cycle) followed by 15 s at 95 °C, 30 s at 60 °C, and 30 s at 72 °C (40 cycles). Primers for the transcript analyses of conidiation- and appressorium-related genes are listed in Table 1. To measure the relative abundance of each transcript, *Ct* values were normalized to those of *β-*tubulin (MGG_00604) and expressed as 2^*−ΔCt*^, where *−ΔCt* = (C_t, target gene_ − C_t, β-tubulin_). Fold changes in expression during fungal conidiation were calculated as 2^*−ΔΔCt*^, where −*ΔΔCt* = (C_t, target gene_ − C_t, β-tubulin_)_conidiation_ − (C_t, WT_ − C_t, β-tubulin_)_conidiation_^45^. qRT-PCR was performed with three independent pools of tissues in two sets of experimental replicates.

### Developmental phenotype assays

Vegetative growth was measured on OMA, V8, and MM (3% sucrose, 0.2% NaNO_3_, 0.1% KH_2_PO_4_, 0.05% MgSO_4_∙7H_2_O, 0.05% KCl, 0.01% trace element, and 1.5% agar powder) 6 days after inoculation in six-well plates, with three replicates. To measure septum distance, hyphae on CM agar plates were stained with CFW to visualize septa and observed under a light microscope and under UV. The ability to produce conidia was measured by counting the number of conidia in 7-day-old V8 agar six-well plates. Conidia were collected by flooding the plate with 5 ml of sterilized distilled water. Conidia were counted using a hemocytometer under a microscope. Conidial germination and appressorium formation were measured on coverslips (Waldemar Knittel Glasbearbeitungs, Braunschweig, Germany). Harvested conidia were filtered through two layers of Miracloth (Calbiochem, La Jolla, CA, USA). Conidial suspensions (5 × 10^4^ conidia ml^−1^) were placed on the hydrophobic side of the coverslips, placed in a moistened box, and incubated at 25 °C. After germination, conidia were measured. The percentage of germinated conidia forming appressoria was determined by microscopic examination of at least 100 conidia in three independent experiments and in triplicate.

### Plant infection assays

For the spray infection assay, conidia were harvested from 8- to 10-day-old cultures on OMA plates. In total, 10 ml of conidial suspension (5 × 10^5^ conidia ml^−1^) containing 250 ppm Tween 20 was sprayed onto 3-week-old plants of susceptible rice (cv. Nakdongbyoe). The inoculated plants were kept in a dew chamber at 25 °C for 24 h in the dark and moved to a growth chamber with a photoperiod of 16 h with fluorescent lights^46^. Disease severity was measured at 7 days after inoculation. Assays for appressorium penetration and invasive growth were performed using rice sheath tissues as described previously^47^. Briefly, conidial suspension (2 × 10^4^ conidia ml^−1^) was dropped onto rice sheath tissue and incubated in a humid chamber at 25 °C. Invasive infection of hyphae was observed after 48 h by light microscopy.

## Electronic supplementary material

Supplementary information

## References

[1] Dean R (2012). The top 10 fungal pathogens in molecular plant pathology. Mol. Plant Pathol..

[2] Skamnioti P, Gurr SJ (2009). Against the grain: safeguarding rice from rice blast disease. Trends Biotechnol..

[3] Talbot NJ (2003). On the trail of a cereal killer: exploring the biology of *Magnaporthe grisea*. Annu. Rev. Microbiol..

[4] Lee YH, Dean RA (1994). Hydrophobicity of contact surface induces appressorium formation in *Magnaporthe grisea*. FEMS Microbiol. Lett..

[5] Li G, Zhou X, Xu JR (2012). Genetic control of infection-related development in *Magnaporthe oryzae*. Curr. Opin. Microbiol..

[6] Li Y, Zhang X, Hu S, Liu H, Xu JR (2017). PKA activity is essential for relieving the suppression of hyphal growth and appressorium formation by MoSfl1 in *Magnaporthe oryzae*. PLoS Genet..

[7] Jiang C, Zhang X, Liu H, Xu JR (2018). Mitogen-activated protein kinase signaling in plant pathogenic fungi. PLoS Pathog..

[8] Zhao X, Kim Y, Park G, Xu JR (2005). A mitogen-activated protein kinase cascade regulating infection-related morphogenesis in *Magnaporthe grisea*. Plant cell.

[9] Thines E, Weber RW, Talbot NJ (2000). MAP kinase and protein kinase A–dependent mobilization of triacylglycerol and glycogen during appressorium turgor generation by *Magnaporthe grisea*. Plant cell.

[10] Xu JR, Staiger CJ, Hamer JE (1998). Inactivation of the mitogen-activated protein kinase Mps1 from the rice blast fungus prevents penetration of host cells but allows activation of plant defense responses. Proc. Natl. Acad. Sci. USA.

[11] Jeon J (2008). A putative MAP kinase kinase kinase, *MCK1*, is required for cell wall integrity and pathogenicity of the rice blast fungus, *Magnaporthe oryzae*. Mol. Plant Microbe. Interact..

[12] Cools T, De Veylder L (2009). DNA stress checkpoint control and plant development. Curr. Opin. Plant Biol..

[13] Kipreos ET (2005). *C*. *elegans* cell cycles: invariance and stem cell divisions. Nat. Rev. Mol. Cell Biol..

[14] Saunders DG, Aves SJ, Talbot NJ (2010). Cell cycle–mediated regulation of plant infection by the rice blast fungus. Plant cell.

[15] Veneault-Fourrey C, Barooah M, Egan M, Wakley G, Talbot NJ (2006). Autophagic fungal cell death is necessary for infection by the rice blast fungus. Science.

[16] Osés-Ruiz M, Sakulkoo W, Littlejohn GR, Martin-Urdiroz M, Talbot NJ (2017). Two independent S-phase checkpoints regulate appressorium-mediated plant infection by the rice blast fungus *Magnaporthe oryzae*. Proc. Natl. Acad. Sci. USA.

[17] Bardin AJ, Amon A (2001). Men and sin: what’s the difference?. Nat. Rev. Mol. Cell Biol..

[18] Palani S, Meitinger F, Boehm ME, Lehmann WD, Pereira G (2012). Cdc14-dependent dephosphorylation of Inn1 contributes to Inn1–Cyk3 complex formation. J. Cell. Sci..

[19] Li, C. *et al*. *MoCDC14* is important for septation during conidiation and appressorium formation in *Magnaporthe oryzae*. *Mol*. *Plant Pathol*. in press; 10.1111/mpp.12523 (2016). 10.1111/mpp.12523 PMC6638023 27935243

[20] Roxström‐Lindquist K, Faye I (2001). The *Drosophila* gene *Yippee* reveals a novel family of putative zinc binding proteins highly conserved among eukaryotes. Insect Mol. Biol..

[21] Hosono K (2010). YPEL5 protein of the YPEL gene family is involved in the cell cycle progression by interacting with two distinct proteins RanBPM and RanBP10. Genomics.

[22] Hosono K, Sasaki T, Minoshima S, Shimizu N (2004). Identification and characterization of a novel gene family YPEL in a wide spectrum of eukaryotic species. Gene.

[23] Farlie P (2001). Ypel1: a novel nuclear protein that induces an epithelial‐like morphology in fibroblasts. Genes Cells.

[24] Kelley KD (2010). YPEL3, a p53-regulated gene that induces cellular senescence. Cancer Res..

[25] Liang P (2010). MVP interacts with YPEL4 and inhibits YPEL4-mediated activities of the ERK signal pathway. Biochem. Cell Biol..

[26] Lee JY (2017). Pro-apoptotic role of the human *YPEL5* gene identified by functional complementation of a yeast *moh1Δ* mutation. J. Microbiol. Biotechnol..

[27] Jeon J, Rho H, Kim S, Kim KS, Lee YH (2014). Role of *MoAND1*-mediated nuclear positioning in morphogenesis and pathogenicity in the rice blast fungus, *Magnaporthe oryzae*. Fungal Genet. Biol..

[28] Han JH, Lee HM, Shin JH, Lee YH, Kim KS (2015). Role of the *MoYAK1* protein kinase gene in *Magnaporthe oryzae* development and pathogenicity. Environ. Microbiol..

[29] Liu W (2011). Multiple plant surface signals are sensed by different mechanisms in the rice blast fungus for appressorium formation. PLoS Pathog..

[30] Geoghegan IA, Gurr SJ (2016). Chitosan mediates germling adhesion in *Magnaporthe oryzae* and is required for surface sensing and germling morphogenesis. PLoS Pathog..

[31] Choi J, Kim KS, Rho HS, Lee YH (2011). Differential roles of the phospholipase C genes in fungal development and pathogenicity of *Magnaporthe oryzae*. Fungal Genet. Biol..

[32] Rho HS, Jeon J, Lee YH (2009). Phospholipase C-mediated calcium signalling is required for fungal development and pathogenicity in *Magnaporthe oryzae*. Mol. Plant Pathol..

[33] Parsons KA, Chumley FG, Valent B (1987). Genetic transformation of the fungal pathogen responsible for rice blast disease. Proc. Natl. Acad. Sci. USA.

[34] Mulder NJ (2005). InterPro, progress and status in 2005. Nucleic Acids Res..

[35] Kumar S, Stecher G, Tamura K (2016). MEGA7: Molecular evolutionary genetics analysis version 7.0 for bigger datasets. Mol. Biol. Evol..

[36] Yu JH (2004). Double-joint PCR: a PCR-based molecular tool for gene manipulations in filamentous fungi. Fungal Genet. Biol..

[37] Choi J, Kim Y, Kim S, Park J, Lee YH (2009). *MoCRZ1*, a gene encoding a calcineurin-responsive transcription factor, regulates fungal growth and pathogenicity of *Magnaporthe oryzae*. Fungal Genet. Biol..

[38] Leung H (1990). Transformation of the rice blast fungus *Magnaporthe grisea* to hygromycin B resistance. Curr. Genet..

[39] Sweigard JA, Chumley FG, Valent B (1992). Cloning and analysis of CUT1, a cutinase gene from *Magnaporthe grisea*. Mol. Gen. Genet..

[40] Chi MH, Park SY, Lee YH (2009). A quick and safe method for fungal DNA extraction. Plant Pathol. J..

[41] Yi M (2009). The ER chaperone LHS1 is involved in asexual development and rice infection by the blast fungus *Magnaporthe oryzae*. Plant cell.

[42] Sambrook, J. & Russel, D. W. *Molecular Cloning: A Laboratory Manual*. 3rd ed. (Cold Spring Harbor Laboratory Press, 2001).

[43] Horwitz BA (1999). A G protein alpha subunit from *Cochliobolus heterostrophus* involved in mating and appressorium formation. Fungal Genet. Biol..

[44] Kim KS, Lee YH (2012). Gene expression profiling during conidiation in the rice blast pathogen *Magnaporthe oryzae*. PLoS One.

[45] Livak KJ, Schmittgen TD (2001). Analysis of relative gene expression data using real-time quantitative PCR and the 2^−ΔΔC^_T_ method. Methods.

[46] Park J, Kong S, Kim S, Kang S, Lee YH (2014). Roles of forkhead-box transcription factors in controlling development, pathogenicity, and stress response in *Magnaporthe oryzae*. Plant Pathol. J..

[47] Koga H, Dohi K, Nakayachi O, Mori M (2004). A novel inoculation method of *Magnaporthe grisea* for cytological observation of the infection process using intact leaf sheaths of rice plants. Physiol. Mol. Plant Pathol..

[48] Kim S (2009). Homeobox transcription factors are required for conidiation and appressorium development in the rice blast fungus *Magnaporthe oryzae*. PLoS Genet..

[49] Park SY (2013). Global expression profiling of transcription factor genes provides new insights into pathogenicity and stress responses in the rice blast fungus. PLoS Pathog..

[50] Nishimura M (2009). Mstu1, an APSES transcription factor, is required for appressorium-mediated infection in *Magnaporthe grisea*. Biosci. Biotechnol. Biochem..

[51] Zhou Z, Li G, Lin C, He C (2009). *Conidiophore stalk-less1* encodes a putative zinc-finger protein involved in the early stage of conidiation and mycelial infection in *Magnaporthe oryzae*. Mol. Plant Microbe Interact..

[52] Lau GW, Hamer JE (1998). Acropetal: a genetic locus required for conidiophore architecture and pathogenicity in the rice blast fungus. Fungal Genet. Biol..

[53] Madi L, Ebbole DJ, White BT, Yanofsky C (1994). Mutants of *Neurospora crassa* that alter gene expression and conidia development. Proc. Natl. Acad. Sci. USA.

[54] Odenbach D (2007). The transcription factor Con7p is a central regulator of infection‐related morphogenesis in the rice blast fungus *Magnaporthe grisea*. Mol. Microbiol..

[55] Lee BN, Adams TH (1994). The *Aspergillus nidulans fluG* gene is required for production of an extracellular developmental signal and is related to prokaryotic glutamine synthetase I. Genes Dev..

[56] Wieser J, Lee BN, Fondon JW, Adams TH (1994). Genetic requirements for initiating asexual development in *Aspergillus nidulans*. Curr. Genet..

[57] Matheis S (2017). Functions of the *Magnaporthe oryzae* Flb3p and Flb4p transcription factors in the regulation of conidiation. Microbiol. Res..

[58] Xu JR, Urban M, Sweigard JA, Hamer JE (1997). The *CPKA* gene of *Magnaporthe grisea* is essential for appressorial penetration. Mol. Plant Microbe Interact..

[59] Xu JR, Hamer JE (1996). MAP kinase and cAMP signaling regulate infection structure formation and pathogenic growth in the rice blast fungus *Magnaporthe grisea*. Genes Dev..

[60] Choi W, Dean RA (1997). The adenylate cyclase gene *MAC1* of *Magnaporthe grisea* controls appressorium formation and other aspects of growth and development. Plant Cell.

