# Supplementary material for: Distinct roles of the YPEL gene family in development and pathogenicity in the ascomycete fungus Magnaporthe oryzae
Source: Sci Rep. 2018 Sep 27;8:14461. doi: 10.1038/s41598-018-32633-6 (PMC6160453; doi:10.1038/s41598-018-32633-6)
Supplement: Supplementary file 1 — Supplementary information [file 41598_2018_32633_MOESM1_ESM.docx]

**Distinct roles of the *YPEL* gene family in development and pathogenicity in the ascomycete fungus *Magnaporthe oryzae***

**Joon-Hee Han^1^, Jong-Hwan Shin^1^, Yong-Hwan Lee^2^ & Kyoung Su Kim^1,^ ***

^1^Division of Bio-resource Sciences, College of Agriculture and Life Sciences, Kangwon National University, Chuncheon 24341, Korea

^2^Department of Agricultural Biotechnology, College of Agriculture and Life Sciences, Seoul National University, Seoul 08826, Korea

*Corresponding author

Phone +82-33-250-6435, Fax +82-33-259-5558, E-mail kims@kangwon.ac.kr


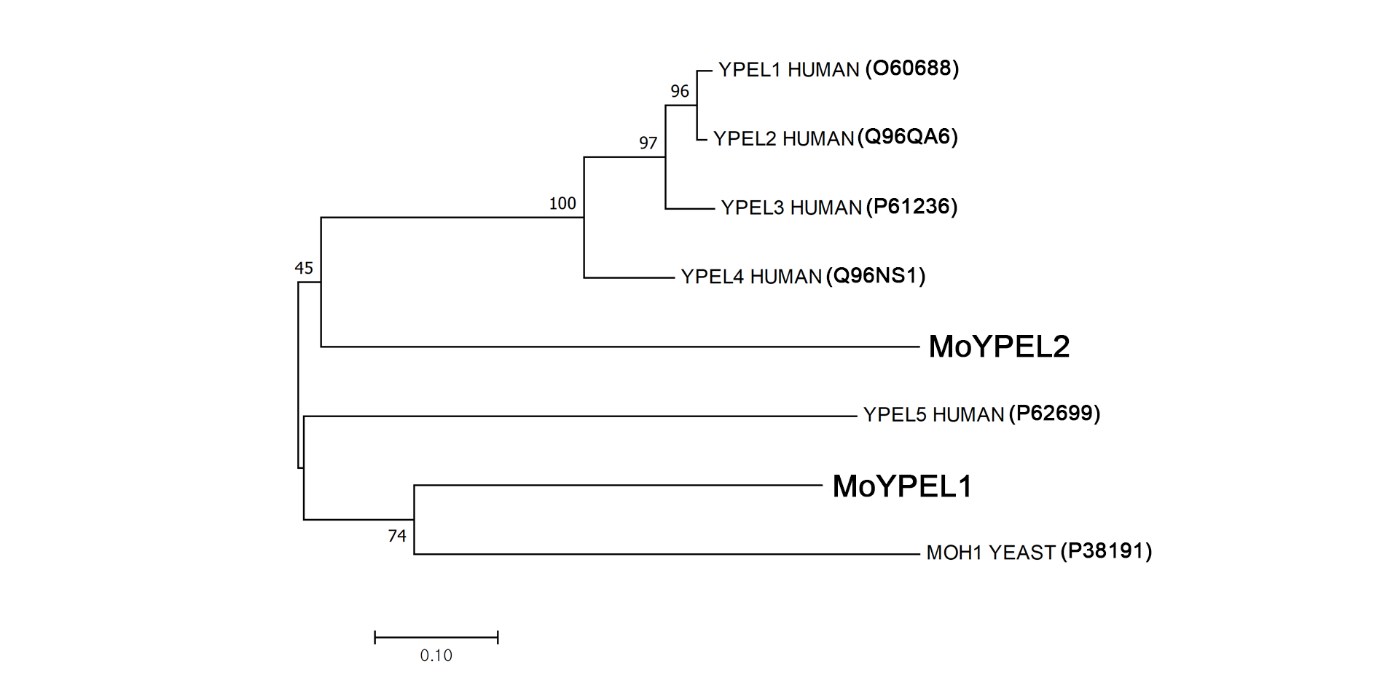


**Figure S1.** Phylogenetic analysis of MoYPEL proteins derived from the alignment of the amino acid sequences of the related proteins. Numbers at nodes represent the percentage of the occurrence in 1,000 bootstrap replicates. Scale bar indicates the number of amino acid differences per site. GenBank accession numbers are in parentheses followed by species.

**
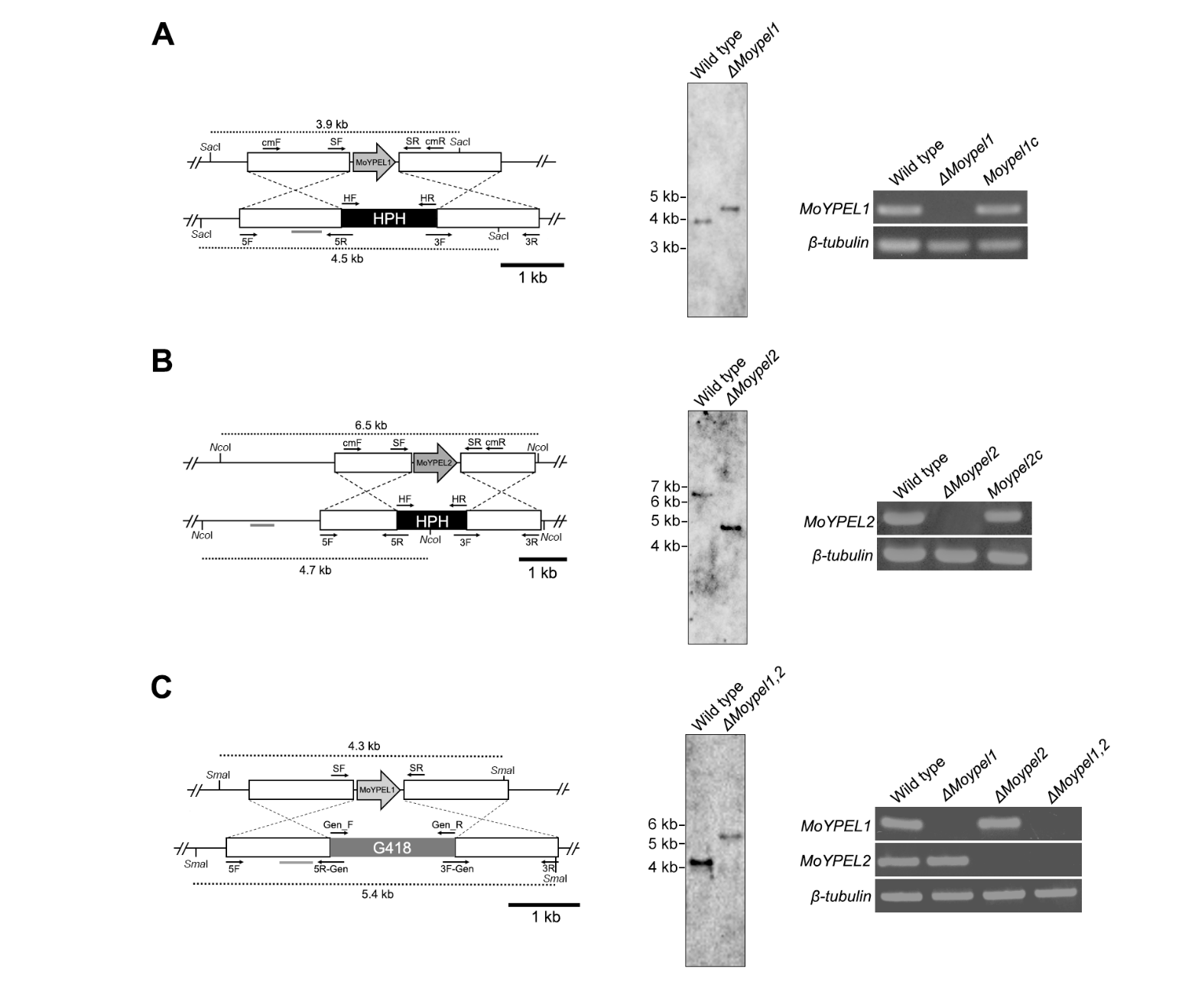
** **Figure S2.** Schematic representation of the targeted deletion of *MoYPEL* genes in *Magnaporthe oryzae*. Double-joint polymerase chain reaction (PCR) was used to generate the construct using the primers listed in Table S2. Southern blot and quantitative reverse transcription (qRT)-PCR were performed to confirm deletion of the *MoYPEL1* gene (A), the *MoYPEL2* gene (B), and both *MoYPEL1* and *MoYPEL2* genes (C). Gray bars represent the regions used for probes. Gel images were cropped from different parts of the same gel using white space for better display.


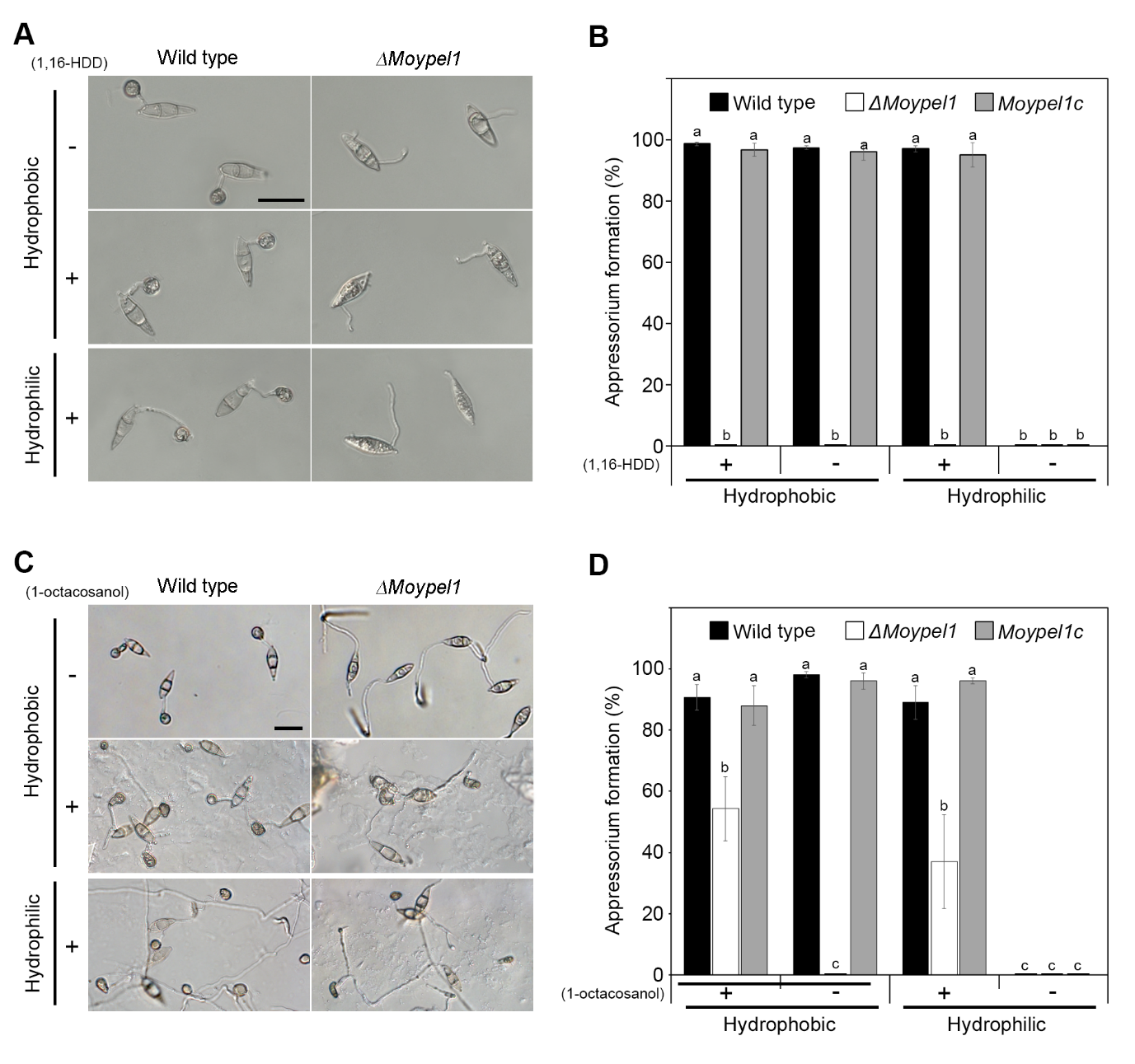
**Figure S3.** Assays for the effect of chemical inducers on appressorium development. A cutin monomer, 1,16-hexadecanediol (10 µM) and a primary alcohol, 1-octacosanol (10 mM) was exogenously amended on both hydrophobic and hydrophilic surfaces, respectively. Appressorium development was then measured 24 h after the treatment. Data were presented as means ± SD from three independent experiments with three replicates per experiment. The letters on bars indicate a significant difference (*P* < 0.05) from three independent experiments determined by Tukey’s test. Scale bars = 30 µm.

**Table S1.** Amino acid identity of compare with MoYPEL and YPEL genes

| Protein | Query cover / identity (%) | | | | | | | | | | | | | | | | | | | | | |
| --- | --- | --- | --- | --- | --- | --- | --- | --- | --- | --- | --- | --- | --- | --- | --- | --- | --- | --- | --- | --- | --- | --- |
|  | Human | | | | | Fungi | | | | | | | | | | | | | | | | |
|  | *YPEL1^*^* | *YPEL2* | *YPEL3* | *YPEL4* | *YPEL5* | *YPEL1* | | | | | | | | | *YPEL2* | | | | | | | |
|  |  |  |  |  |  | *Cg*^‡^ | *Cs* | *Gf* | *Nc* | *An* | *Um* | *Pg* | *Sc* | *Pi* | *Um* | *Pi* | *An* | *Nc* | *Gf* | *Cg* | *Cs* |  |
| MoYPEL1 | 56/52 | 56/51 | 56/51 | 57/48 | 61/42 | 63/88 | 63/89 | 63/85 | 63/82 | 63/78 | 63/57 | 63/51 | 55/49 | 59/54 | 51/44 | 53/40 | 71/39 | 51/35 | 53/37 | 52/32 | 52/33 |  |
| MoYPEL2 | 40/40 | 40/39 | 40/40 | 41/42 | 38/34 | 34/36 | 34/36 | 34/39 | 34/40 | 40/36 | 35/37 | 36/41 | 37/37 | 41/40 | 45/33 | 36/40 | 74/41 | 80/36 | 71/43 | 39/55 | 68/39 |  |

^*^Accession number of human *YPEL* genes are YPEL1 (O60688), YPEL2 (Q96QA6), YPEL3 (P61236), YPEL4 (Q96NS1), and YPEL5 (P62699).

^‡^Homologues of MoYPEL were found in the genomes of the following species: *Cg* (*Colletotrichum graminicola*), *Cs* (*Colletotrichum scovillei*), *Gf* (*Gibberella fujikuroi*), *Nc* (*Neurospora crassa*), *An* (*Aspergillus niger*), *Um* (*Ustilago maydis*), *Pg* (*Puccinia graminis*), *S c*(*Saccharomyces cerevisiae*), and *Pi* (*Phytophthora infestans*). Accession number are shown in Figure 1.

**Table S2.** Phenotypic characterization of transformants used in this study

| Strains | Growth length  (mm)^a^ | Distance of septum  (µm)^b^ | Conidiation  (×10^4^/ml)^c^ | Germination  (%)^d^ | Appressorium formation (%)^e^ |
| --- | --- | --- | --- | --- | --- |
| Wild-type | 38.3 ± 1.5^Af^ | 135.5 ± 8.1^A^ | 48.7 ± 3.1^B^ | 98.0 ± 1.0^A^ | 96.3 ± 1.2^A^ |
| *ΔMoypel1* | 30.0 ± 1.0^B^ | 29.3 ± 8.8^B^ | 10.7 ± 4.0^C^ | 89.7 ± 1.5^B^ | ND^g^ |
| *Moypel1c* | 38.7 ± 1.5^A^ | 111.5 ± 15.2^A^ | 43.7 ± 6.7^B^ | 96.7 ± 0.6^A^ | 95.7 ± 2.5^A^ |
| *ΔMoypel2* | 36.3 ± 1.5^A^ | 118.9 ± 15.0^A^ | 95.3 ± 6.4^A^ | 98.3 ± 0.6^A^ | 98.0 ± 1.0^A^ |
| *Moypel2c* | 37.0 ± 1.0^A^ | 120.5 ± 20.1^A^ | 52.7 ± 5.9^B^ | 98.7 ± 0.6^A^ | 98.3 ± 0.6^A^ |
| *ΔΔMoypel1,2* | 36.0 ± 2.0^A^ | 131.4 ± 26.0^A^ | 13.3 ± 1.5^C^ | 97.3 ± 0.6^A^ | 96.7 ± 0.6^A^ |
| MoYPEL1:sGFP | 35.3 ± 0.6^A^ | 125.6 ± 14.3^A^ | 42.3 ± 2.5^B^ | 97.0 ± 1.0^A^ | 95.7 ± 0.6^A^ |
| MoYPEL2:sGFP | 38.3 ± 1.5^A^ | 131.4 ± 16.2^A^ | 49.3 ± 6.7^B^ | 97.0 ± 2.0^A^ | 95.7 ± 1.2^A^ |
| H1:RFP | 38.1 ± 1.0^A^ | 125.9 ± 6.5^A^ | 50.2 ± 5.7^B^ | 96.7 ± 1.0^A^ | 96.3 ± 1.2^A^ |

^a^Mycelia growth was measured at 6-day post-inoculation on CM agar media.

^b^Distance of septum was determined by counting at least 50 hyphal compartments per strain.

^c^Conidiation was measured by counting the number of conidia collected with 5 ml of sterilized distilled water from 7-day-old V8 juice agar plates.

^d^Conidial germination on hydrophobic surfaces was measured under a light microscope using conidia harvested from 7-day-old V8 juice agar plates.

^e^Appressorium formation on a hydrophobic slide surface in humid box was counted that DIC using conidia harvested from 7-day-old V8 juice agar plates.

^f^Data were presented as means±SD from three independent experiments. Different letters indicate significant differences according to Tukey’s test at *p* < 0.05.

^g^ND = not determined.

**Table S3.** Primers used in this study

| Primers | Sequence (5’ → 3’) | |
| --- | --- | --- |
| **MoYPEL1** | | |
| 5F | AGTTCACACACACGCACACA | |
| 5R | CCTCCACTAGCTCCAGCCAAGCCTGACAAGGCAAGGTAAGCAGGT | |
| 3F | GTTGGTGTCGATGTCAGCTCCGGAGCACTACTGCAAAGCAAGGGT | |
| 3R | TTATCTTCCGGCAGACGCCATT | |
| NF | GCGGCTTGTGGTGCATATTGTA | |
| NR | CGAAGCACTCACCAGGCCA | |
| SF | ACTCAAGTCACACCACCAAACC | |
| SR | ATCGGGTGTAACAGGCTAGT | |
| PF | AAGCAGCCTTACCAACCAACCAAC | |
| PR | TGGACCGGTGTGCTATTCGTTTCT | |
| cmF | TCTTTCTATCCGCATGACTCTCGG | |
| cmR | TCCAAAGCATCAGCTACCGT | |
| RTF | TCGGCTGCAAGAACTGCAAA | |
| RTR | ATACACTGCACAGCAACTCG | |
| qRTF | TCGTAACTTCCGCGGTCAACAT | |
| qRTR | TCCTTGCACTGCTTGCACAT | |
| **MoYPEL2** | | |
| 5F | TCTCCTTCTCCAGCGAACGAACCA | |
| 5R | CCTCCACTAGCTCCAGCCAAGCCTCCTGAAGGAGAACCACAGCTTGA | |
| 3F | GTTGGTGTCGATGTCAGCTCCGGAGTGTAAGATACCCTGGGATGGAGGA | |
| 3R | ATAACGCCCGGGATCTGGATGTTG | |
| NF | GACCGGGCTGTCTGATTTGTGTTT | |
| NR | TGCCGAGCCAGTAGTTGTCTGATA | |
| SF | TTAAGATCGAGCCGTTTGGCTTGG | |
| SR | TGTCTGGCTGTGGGTTGTTACAGT | |
| PF | ATGGCAGCAACCAACCTGCAAT | |
| PR | ATCGCGCATCAGGGAAGAATGT | |
| cmF | AAGATCGAGCCGTTTGGCTT | |
| cmR | ATCCTCCATCCCAGGGTATCTT | |
| RTF | TGGTCAACGTCAGCATCGGG | |
| RTR | TTCGCAGCATCAAGCTCCATC | |
| qRTF | AAATTCATCCTCGAGACGTCGCAC | |
| qRTR | AACAGCTCATCGCACTCATCCT | |
| **MoYPEL1,2** | | |
| 3F-Gen | GCACAGGTACACTTGTTTAGAGCACTACTGCAAAGCAAGGGT | |
| 5R-Gen | CCTTCAATATCATCTTCTTGACAAGGCAAGGTAAGCAGGT | |
| **Hygromycin phosphotransferase** | | |
| HPHF | GGCTTGGCTGGAGCTAGTGGAGG | |
| HPHR | CTCCGGAGCTGACATCGACACCAAC | |
| **G418** | | |
| Gen_F | AGAAGATGATATTGAAGG | |
| Gen_R | CTCTAAACAAGTGTACCTGTGC | |
| **β-tublin** | | |
| β -tubF | TCGACAGCAATGGAGTTTAC | |
| β -tubR | AGCACCAGACTGACCGAAGAC | |
| **YPEL1:GFP** | | |
| YPEL1_F | ACTGACAAGCAAAAGAAGCCAG | |
| YPEL1_R | AGATACACTGCACAGCAACTC | |
| pIG-YPEL1_F | CTGTGCAGTGTATCTATGGTGAGCAAGGGCGAGG | |
| pIG-YPEL1_R | CTTTTGCTTGTCAGTCAACATACGAGCCGGAAGC | |
| **YPEL2:GFP** | | |
| YPEL2_F | GGCCACCTCGCCGTAACTAC | |
| YPEL2_R | TCTCCTGGCCTTCGCAGCAT | |
| pIG-YPEL2_F | GCGAAGGCCAGGAGAATGGTGAGCAAGGGCGAGG | |
| pIG-YPEL2_R | TACGGCGAGGTGGCCCAACATACGAGCCGGAAGC | |
| **qRT-PCR** | | |
| *MoHOX2* | qRTF | TGGGGTTCTGCAGCCATGTT |
|  | qRTR | GTCCCGTGGTGTTACGTTCTGG |
| *MoHOX7* | qRTF | CGGACGGCTCCAAGATTCTCC |
|  | qRTR | CTGCCACGCTTCATGCCAA |
| *MoPLC1* | qRTF | GCCCTCTACCGACACAACAT |
|  | qRTR | TAGCCCGACTGATCCATACC |
| *MoPLC2* | qRTF | CGAGAAGTATGGGCAAGAGC |
|  | qRTR | CCTGCAAACATCCCTTCATT |
| *MoPLC3* | qRTF | TTTCGACTCGTCCAACTT |
|  | qRTR | GAGTCGCTGCTTCTGTACCC |
| *MoAPS1* | qRTF | TAGCAACCATCAACGCAACGACC |
|  | qRTR | GCACACGGCAAAGTGTCGTTGCGCTCAA |
| *MoAPS2* | qRTF | CGGCACGGGCGAGAAGA |
|  | qRTR | TTTACCATGCCATCCGACACCT |
| *Mstu1* | qRTF | GATGACCGTTCGAGCAGCAGTG |
|  | qRTR | GCGGGGCGGTTCATTGTC |
| *COS1* | qRTF | CTCAGCCCACATACAACTACCAGC |
|  | qRTR | GACGACGATGATGATGGCGATG |
| *MoCRZ1* | qRTF | CCTGCATTCTGGAGAGAAG |
|  | qRTR | AGATTCTTTGCCGTTCCAT |
| *ACR1* | qRTF | CGACATGCTCAGCTCTTACAGG |
|  | qRTR | TAGTCGTATCGCTCCGTGCCAT |
| *MoCON6* | qRTF | CACAAGGCCAACCTCAA |
|  | qRTR | TCTCCATCTCCTCGAGAC |
| *CON7* | qRTF | CCAGGCTGGTTCGGATGTATCTC |
|  | qRTR | CCTGGTTGGACCCTCCGT |
| *MoCON8* | qRTF | GATTCATCCGCAGCAAA |
|  | qRTR | CACATCTTGCCAAACAGG |
| *MoFLUG* | qRTF | AGCTCGAGATTCCTTGCCCA |
|  | qRTR | CAATCCGTTACAGCCTCATCCC |
| *MoFLBA* | qRTF | AGCGCTGGATGGAGTCAAAGAG |
|  | qRTR | TACGGCGTTTTGTGCATCCTC |
| *Flb3* | qRTF | GACCAAGTTCGCTCCAGACG |
|  | qRTR | GTCCTCGGAGCCAGCCT |
| *Flb4* | qRTF | CCAGATCCATCAACGCCGC |
|  | qRTR | TCAAGCACGGAAGCGACTCG |
| *MCK1* | qRTF | ACTGCTTCACAGTCGATTCC |
|  | qRTR | CCGAGTCAAGGAAGTTGTAGTT |
| *CPKA* | qRTF | CTACACGCCAAAAACATCAT |
|  | qRTR | CAGACTTGTTGTAGCCCTTG |
| *PMK1* | qRTF | TTGGACCTGTTGGAGAAGTTG |
|  | qRTR | CATGGTACGGCTCAAGGTAAG |
| *MAC1* | qRTF | AGACTTACCAGGGAGCAAAC |
|  | qRTR | ATACACCACCTCTGGGTTCT |
